# The glycoprotein CD147 defines miRNA‐enriched extracellular vesicles that derive from cancer cells

**DOI:** 10.1002/jev2.12318

**Published:** 2023-03-27

**Authors:** Song Yi Ko, WonJae Lee, Melanie Weigert, Eric Jonasch, Ernst Lengyel, Honami Naora

**Affiliations:** ^1^ Department of Molecular and Cellular Oncology University of Texas MD Anderson Cancer Center Houston Texas USA; ^2^ Section of Gynecologic Oncology Department of Obstetrics and Gynecology University of Chicago Chicago Illinois USA; ^3^ Department of Genitourinary Medical Oncology University of Texas MD Anderson Cancer Center Houston Texas USA

**Keywords:** cancer, extracellular vesicles, liquid biopsy, miRNA, surface marker

## Abstract

Extracellular vesicles (EVs) are ideal for liquid biopsy, but distinguishing cancer cell‐derived EVs and subpopulations of biomarker‐containing EVs in body fluids has been challenging. Here, we identified that the glycoproteins CD147 and CD98 define subpopulations of EVs that are distinct from classical tetraspanin^+^ EVs in their biogenesis. Notably, we identified that CD147^+^ EVs have substantially higher microRNA (miRNA) content than tetraspanin^+^ EVs and are selectively enriched in miRNA through the interaction of CD147 with heterogeneous nuclear ribonucleoprotein A2/B1. Studies using mouse xenograft models showed that CD147^+^ EVs predominantly derive from cancer cells, whereas the majority of tetraspanin^+^ EVs are not of cancer cell origin. Circulating CD147^+^ EVs, but not tetraspanin^+^ EVs, were significantly increased in prevalence in patients with ovarian and renal cancers as compared to healthy individuals and patients with benign conditions. Furthermore, we found that isolating miRNAs from body fluids by CD147 immunocapture increases the sensitivity of detecting cancer cell‐specific miRNAs, and that circulating miRNAs isolated by CD147 immunocapture more closely reflect the tumor miRNA signature than circulating miRNAs isolated by conventional methods. Collectively, our findings reveal that CD147 defines miRNA‐enriched, cancer cell‐derived EVs, and that CD147 immunocapture could be an effective approach to isolate cancer‐derived miRNAs for liquid biopsy.

## 1 INTRODUCTION

Extracellular vesicles (EVs) hold great potential for liquid biopsy because EVs can be detected in body fluids and protect their cargo from degradation (Maas et al., 2017; Xu et al., 2018). Because the EV cargo often reflects the genetic and biological status of the parental cell, constituents of EV cargo have been extensively investigated as potential cancer biomarkers (Hannafon et al., 2016; Shin et al., 2021; Wang et al., 2022; Xu et al., 2018; Zhou et al., 2014). However, almost all types of cells release EVs and it is unclear what proportion of EVs in body fluids of cancer patients derive from cancer cells. Circulating EVs are elevated in other pathologic conditions such as diabetes and hypertension (Li et al., 2016; Preston et al., 2003) that are common comorbidities of cancer patients (Roy et al., 2018). Cancer cell‐derived EVs might constitute only a minor fraction of EVs in body fluids of cancer patients who have comorbid conditions and/or small tumours. As such, detecting biomarkers in these EVs could be very difficult. Currently, there are no well‐defined methods that can identify cancer cell‐derived EVs in body fluids. The tetraspanins CD63, CD81 and CD9 are commonly used as surface markers of EVs, but are ubiquitously expressed (Maecker et al., 1997) and cannot differentiate EVs that are released by cancer cells and by normal cells. It has been reported that the cell surface proteoglycan glypican‐1 is enriched in circulating EVs of patients with pancreatic cancer (Melo et al., 2015). However, glypican‐1 is expressed in the stroma as well as in pancreatic cancer cells, and has been detected in EVs released by stromal fibroblasts (Nigri et al., 2022; Tsujii et al., 2021). Developing approaches that can distinguish cancer cell‐derived EVs in body fluids is therefore critical to improve detection of biomarkers contained in these EVs.

Another important consideration for evaluating biomarkers contained in EVs is that an individual cell type, including cancer cells, can release several subpopulations of EVs that vary in their cargo (Kowal et al., 2016). EVs have been captured from body fluids by using antibodies to CD63, CD81 or CD9 (Campos‐Silva et al., 2019; Duijvesz et al., 2015; Logozzi et al., 2009). However, there is considerable evidence that these tetraspanins are unevenly distributed across EVs (Han et al., 2021; Mathieu et al., 2021; Tian et al., 2018). Furthermore, EVs that express at least one tetraspanin have been found to constitute <60% of total EVs in cancer cell‐conditioned media and body fluids (Mizenko et al., 2021; Tian et al., 2018). Tetraspanin‐negative EVs have not been well‐characterized. However, glioblastoma cells have been shown to release epidermal growth factor receptor variant III in large EVs that lack CD63 and CD81 (Yekula et al., 2019) and platelet‐derived growth factor receptor‐α in EVs that lack all three tetraspanins (Lee et al., 2018). These studies highlight the importance of investigating tetraspanin‐negative EVs as a source of cancer biomarkers.

MicroRNAs (miRNAs) contained in EVs have attracted substantial attention as candidate biomarkers for cancer diagnosis, prognosis and recurrence because miRNA expression patterns are frequently dysregulated in cancer (Hannafon et al., 2016; Shin et al., 2021; Wang et al., 2022; Xu et al., 2018; Zhou et al., 2014). However, the miRNA content of EVs has been contentious. On one hand, there are currently more than 10,000 EV‐associated miRNA entries in the Vesiclepedia database, a compendium of biomolecules identified in EVs (Kalra et al., 2012). On the other hand, stoichiometric analysis of the miRNA content in EVs and functional studies have revealed that the majority of EVs do not contain miRNA copy numbers that are biologically significant (Albanese et al., 2021; Chevillet et al., 2014; Zhang et al., 2021). An implication of these studies is that there could exist a subpopulation of EVs that is selectively enriched in miRNA, but this subpopulation has hitherto not been identified.

Here, we show that the glycoprotein CD147 defines a subpopulation of EVs that is distinct from tetraspanin^+^ EVs in its biogenesis, content and cellular origin. In contrast to tetraspanin^+^ EVs, CD147^+^ EVs are not generated by the Endosomal Sorting Complex Required for Transport (ESCRT) machinery, and are selectively enriched in miRNA through the interaction of CD147 with the miRNA‐binding protein heterogeneous nuclear ribonucleoprotein A2/B1 (hnRNP A2/B1). Furthermore, in contrast to tetraspanin^+^ EVs, CD147^+^ EVs predominantly derive from cancer cells and increase in prevalence in cancer patients from early stages of disease. Moreover, isolating circulating miRNAs by CD147 immunocapture, as compared to conventional methods, increases the sensitivity of detecting cancer cell‐specific miRNAs and yields miRNAs that more closely reflect the tumour miRNA signature. Because CD147 is overexpressed in many types of cancer, CD147 immunocapture could be used to detect cancer‐derived circulating miRNAs in multiple disease sites.

## 2 MATERIALS AND METHODS

### 2.1 Antibodies and plasmids

Antibodies used in this study are described in Table S1. Plasmids for expressing CD63‐GFP, CD81‐GFP, CD9‐GFP, CD147‐GFP and CD98‐GFP fusion proteins were as follows: CD63‐pEGFP C2 (gift from Paul Luzio, University of Cambridge; Addgene #62964), mEmerald‐CD81‐10 (He et al., 2013), mEmerald‐CD9‐10 (gifts from Michael Davidson, Florida State University; Addgene #54031, #54029), pCMV3‐CD147‐GFPSpark and pCMV3‐CD98‐GFPSpark (purchased from Sino Biological Inc.; HG10186‐ACG, HG16415‐ACG). Other plasmids were as follows: tetracycline‐regulated miR‐302 cluster expression plasmid pCW57‐GFP‐miR‐302 (Peskova et al., 2019) (gift from Tomáš Bárta, Masaryk University; Addgene #132549), LSB‐hsa‐miR‐302a‐3p and LSB‐hsa‐miR‐302c‐3p reporter plasmids (Gam et al., 2018) (gifts from Ron Weiss, Massachusetts Institute of Technology; Addgene #103400, #103403), TRIPZ HGS shRNA, TRIPZ TSG101 shRNA and TRIPZ vector (purchased from Horizon Discovery Biosciences; RHS4740‐EG9146, RHS4740‐EG7251, RHS4750).

### 2.2 Cell culture

Parental 293T, HeLa, SKOV3 and 786‐O cell lines, normal primary human renal proximal tubule epithelial cells (RPTEC), and normal primary human umbilical vein endothelial cells (HUVEC) were purchased from American Type Culture Collection. The ID8 cell line was provided by Katherine Roby (University of Kansas). Sources of cell lines that stably express the tetracycline repressor protein were as follows: T‐Rex™−293T, T‐Rex™‐HeLa (Invitrogen) and T‐Rex™‐SKOV3 (Applied Biological Materials Inc.). The 293T cell line in which the *HNRNPA2B1* gene was deleted by CRISPR/Cas9 gene editing was purchased from Abcam. All cell stocks were confirmed to be free of mycoplasma contamination and authenticated by short tandem repeat analysis. Culture media was purchased from Corning. Cell lines were cultured in Dulbecco's Modified Eagle Medium (293T, 786‐O, ID8), RPMI 1640 (HeLa) or McCoy's 5A medium (SKOV3) supplemented with 10% FBS (for parental lines) or Tet‐approved FBS (for T‐Rex™ lines), 100 units/mL penicillin and 100 μg/mL streptomycin. RPTEC were cultured in Dulbecco's Modified Eagle Medium/Ham's F‐12 medium (1:1) supplemented with 10% FBS, 100 units/mL penicillin and 100 μg/mL streptomycin. HUVEC were cultured in Medium 199 supplemented with 20% FBS, Endothelial Cell Growth Supplement (EMD Millipore), 100 units/mL penicillin and 100 μg/mL streptomycin.

### 2.3 Cell transfection

Stably transfected cell lines were generated by transfecting cells using Lipofectamine™ 3000 reagent (Invitrogen) as follows. Parental 293T cells were transfected with marker‐GFP fusion expression plasmids, followed by sorting of GFP‐expressing cells using a BD FACSAria™ II cell sorter. T‐Rex™−293T cells were transfected with TRIPZ vector, TRIPZ HGS shRNA or TRIPZ TSG101 shRNA expression plasmids, followed by selection with 0.5 μg/mL puromycin (Sigma‐Aldrich). Expression of shRNA was induced by adding 1 μg/mL doxycycline (Sigma‐Aldrich) to culture media. Donor cells for assaying EV‐mediated transfer of miR‐302 were generated by transfecting cells of T‐Rex™‐lines with miR‐302 cluster expression plasmid, followed by sorting of GFP‐expressing cells. miR‐302 expression was induced in donor cells by adding 1 μg/mL doxycycline to culture media. Recipient cells were generated by transfecting parental 293T cells with miR‐302a or miR‐302c reporter constructs, followed by sorting of mKate2‐expressing cells.

### 2.4 Clinical specimens

Studies using human tissue specimens were reviewed and approved by the Institutional Research Board of the University of Texas MD Anderson Cancer Center and Institutional Research Board of the University of Chicago. All tissue specimens were residual and received full informed consent for research use from all human subjects. Specimens of tumour tissue, ascites and plasma of patients with ovarian carcinoma, and plasma of patients with benign gynaecologic conditions, were obtained from the Ovarian Cancer Tumour Bank at the University of Chicago and from the Southern Division of the National Cancer Institute‐supported Cooperative Human Tissue Network (CHTN) at Duke University. CA125 levels in plasma samples were measured by using the CA125 Quantikine ELISA Kit (R&D Systems). Specimens of tumour tissue and plasma of patients with renal cell carcinoma were obtained from the Eckstein Tissue Acquisition Laboratory at the University of Texas MD Anderson Cancer Center and from CHTN. Clinicopathologic features of cases are described in Table S2. Plasma was isolated using EDTA‐treated tubes from independent batches of peripheral blood of healthy adult volunteers that were obtained from the University of Texas MD Anderson Cancer Center Blood Bank. Each batch contained pooled blood from different donors. Analysis of EVs in specimens of body fluids was performed blinded to clinical data.

### 2.5 Animal studies

Animal studies were reviewed and approved by the Institutional Animal Care and Use Committee of the University of Texas MD Anderson Cancer Center. Four‐week‐old female nude mice (purchased from Envigo) were inoculated s.c. with 2 × 10^6^ T‐Rex™‐HeLa cells that express the miR‐302 cluster, or with 5 × 10^6^ parental 786‐O cells. To induce miR‐302 in T‐Rex™‐HeLa cells, mice were fed pellets containing doxycycline (200 mg per kg of diet) (Bio‐Serv). Tumour diameters were measured with callipers twice a week, and tumour volume was calculated from two perpendicular measurements. Blood samples (200 μL) were collected retro‐orbitally using EDTA‐treated tubes prior to cancer cell injection, when tumours first become palpable, and every 2 weeks thereafter until tumours reached a volume of ∼1000 mm^3^. Animals were then euthanized by CO_2_ asphyxiation. Platelet‐depleted plasma samples were obtained by centrifugation for 15 min at 2000 x *g*. The only mice that were excluded from analysis were those that did not form tumours.

### 2.6 Isolation of EVs

EVs were isolated from conditioned media, plasma and ascites as previously reported (Ko et al., 2019). In compliance with current guidelines of the International Society for Extracellular Vesicles (Théry et al., 2018), full details are provided. Cells were cultured in media containing 2% FBS for 48 h to generate conditioned media. Conditioned media and body fluids were centrifuged at 2400 x *g* at 4°C for 10 min to remove cells and cell debris, and then concentrated by using a Centricon® Plus‐70 centrifugal filter unit with an Ultracel® 100 kDa cutoff filter (Millipore) to remove soluble proteins and particulates of <100 kD in size. Each concentrated supernatant was mixed with 1.5 mL of Optiprep™ stock solution (60% (w/v) aqueous iodixanol, Axis‐Shield PoC) and placed on the bottom of a 14 × 95 mm polyallomer ultracentrifuge tube (Beckman Coulter). Iodixanol solutions were prepared for the discontinuous gradient by diluting OptiPrep™ stock solution in buffer containing 0.25 M sucrose, 10 mM Tris‐HCl (pH 7.4) and 1 mM EDTA. The gradient was formed by layering iodixanol solutions in the following order: 3.0 mL of 40% solution, 2.5 mL of 20% solution, 2.5 mL of 10% solution, and 2.0 mL of 5% solution. Centrifugation was performed at 200,000 x *g* in a SW 40 Ti rotor (Beckman Coulter) at 4°C for 18 h. Ten gradient fractions of 1.0 mL were collected from the top to bottom. The density of each fraction was determined from absorbance readings at 244 nm using a standard curve of serial dilutions of iodixanol solution (Schröder et al., 1997). Individual fractions were washed with phosphate‐buffered saline (PBS), concentrated by using an Amicon® Ultra‐4 centrifugal filter unit with an Ultracel® 100 kDa cutoff filter (Millipore), and then suspended in PBS for further analysis or purification. To purify EVs that express a given surface marker, 10 μg of biotinylated antibody to the marker was incubated with 100 μL of streptavidin‐conjugated magnetic beads (Invitrogen) at 4°C for 16 h, and washed with PBS. Antibody‐conjugated magnetic beads were then incubated with EVs (∼1 × 10^8^) for 16 h at 4°C. Following magnetic separation, supernatants containing marker‐negative EVs were collected for further analysis. Pellets containing marker‐positive EVs were washed three times with PBS, and then processed as described below to isolate protein or RNA.

### 2.7 Flow cytometry

Acquisition and analysis of flow cytometry data were performed using a BD FACSCanto™ II cytometer equipped with FACS Diva™ software (BD Biosciences). Concentrations of antibodies used are listed in Table S1. To detect cell surface proteins, cells were suspended in PBS containing 1% bovine serum albumin (BSA), and incubated with FITC‐conjugated antibody or isotype control at 4°C for 30 min. Thereafter, cells were washed with PBS containing 1% BSA, fixed in 4% paraformaldehyde, and acquired. Staining was evaluated in the gated population of viable singlet cells. A minimum of 10,000 events was analysed for each sample. Three independent experiments were performed to verify expression of a given surface protein in each cell type. Settings to detect EVs were optimized by using bead calibration kits (100, 200, 500 and 760 nm diameter beads, Bangs Laboratories). Unless indicated otherwise, the absolute number of EVs in a given sample was calculated by using 760 nm beads as counting beads and multiplying the ratio of EV events to beads events with the number of beads in the sample. To detect EV surface proteins, 100 μL of EV sample (∼2 × 10^6^ EVs) was incubated with FITC‐conjugated antibody or isotype control at room temperature (RT) for 30 min. Following incubation, samples were diluted to a final volume of 500 μL in PBS and acquired. Staining was evaluated in the gated population of singlet EVs. A minimum of 10,000 events was analysed for each sample. Three independent experiments were performed to verify expression of a given surface protein in EVs derived from each cell type, where each experiment used a different batch of EVs. Contour and histogram plots were generated by using FlowJo™ software (FlowJo, LLC).

### 2.8 Particle size analysis and immunogold labelling

Particle size distribution of purified EVs was analysed by using a ZetaView® QUATT instrument (Particle Metrix) by Alpha Nano Tech LLC. For each batch purification of EVs, an average of 2 × 10^11^ vesicles was isolated and 10 replicate measurements were made. EV markers were detected by immunogold labelling as previously reported (Ko et al., 2019). Briefly, carbon‐coated, formvar‐coated nickel grids (200 mesh) were treated with poly‐L‐lysine for 30 min. EVs were fixed with 2% paraformaldehyde, loaded onto grids, and allowed to absorb at RT for 1 h. Grids were then placed into PBS containing 2% BSA and 0.1% saponin for 20 min, followed by incubation with primary antibody at 4°C for 16 h. Control grids were incubated without primary antibody. Grids were then rinsed with PBS and floated on drops of secondary antibody conjugated to 10 nm gold particles at RT for 2 h. Concentrations of antibodies used are listed in Table S1. Following incubation, grids were washed with PBS, fixed in 1% glutaraldehyde for 5 min, and washed in H_2_O. Grids were stained for contrast for 1 min with 1% uranyl acetate and allowed to dry. Samples were evaluated under a JEM 1010 transmission electron microscope (JEOL USA, Inc.) at an accelerating voltage of 80 Kv. Images were captured using the AMT Imaging System (Advanced Microscopy Techniques Corp.).

### 2.9 Immunoprecipitation

Cells were lysed with immunoprecipitation buffer (1% Triton X‐100, 25 mM HEPES, 150 mM NaCl, 5 mM MgCl_2_) containing protease inhibitor cocktail (Thermo Fisher Scientific). Cell lysates (500 μg) were then incubated with 10 μg of antibody‐conjugated agarose beads at 4°C for 16 h. Thereafter, beads were washed two times with immunoprecipitation buffer and three times with PBS. The pellets were dissolved in 2X Tris‐glycine sodium dodecyl sulphate (SDS) sample buffer (Thermo Fisher Scientific), and analysed by immunoblot.

### 2.10 Immunoblot analysis

Proteins were extracted by lysing cells and EVs in M‐PER buffer (Thermo Fisher Scientific). Protein concentrations of lysates were determined by Bradford assay (BioRad). Lysates were electrophoresed on SDS‐polyacrylamide gels and then transferred to polyvinylidene difluoride membrane (GE Healthcare). Membranes were blocked with 5% non‐fat milk in Tris‐buffered saline with 0.1% Tween‐20 (TBS‐T) at RT for 1 h and then incubated with primary antibody at 4˚C for 16 h. Following washing with TBS‐T buffer, membranes were incubated with HRP‐conjugated secondary antibody at RT for 45 min, washed, and visualized with ECL detection reagent (Millipore). Concentrations of antibodies used are listed in Table S1. Immunoblot data was verified in three independent experiments.

### 2.11 Immunocytochemistry

Cells were plated in chamber slides at sub‐confluence and allowed to adhere. Thereafter, cells were fixed with 4% formaldehyde on ice for 20 min, and then permeabilized with 0.1% Triton X‐100 in PBS on ice for 20 min. Cells were rinsed three times with PBS, blocked with 1% goat serum in PBS for 30 min, and then incubated for 16 h with FITC‐conjugated antibody. Concentrations of antibodies used are listed in Table S1. Following washing with PBS, cells were stained with 4,6‐diamidino‐2‐phenylindole (DAPI) (Sigma‐Aldrich). Cells were viewed and photographed under a LSM 710 confocal microscope (Zeiss) using ZEN® software (Zeiss).

### 2.12 Analysis of EV‐mediated miRNA transfer

EVs were isolated as described above from parental cell lines and donor cells that express the miR‐302 cluster and were treated with 0.2 μg/mL of RNase A (Thermo Fisher Scientific) for 30 min at 37°C to remove external RNA. Recipient 293T cells that express miR‐302a and miR‐302c reporter constructs were seeded at 2 × 10^4^ cells per well in black 96‐well Optical‐Bottom plates (Thermo Fisher Scientific). Recipient cells were incubated without or with the addition of EVs (∼2 × 10^6^) at 37°C for 48 h. As positive controls, recipient cells were transfected with miR‐302a or miR‐302c mimetics (Sigma‐Aldrich) (1 × 10^4^ copy number) by using Lipofectamine 3000 reagent. Following incubation, recipient cells were washed with PBS. Fluorescence intensities of the far‐red fluorescent protein mKate2 and the blue fluorescent protein EBFP2 were measured in recipient cells using a Spark® microplate reader (Tecan). Activity of each miRNA was calculated as mKate2 intensity relative to EBFP2 intensity. Three independent experiments were performed for each assay, where each experiment used a different batch of EVs.

### 2.13 Analysis of EV uptake

EVs were isolated as described above from 293T cells that express marker‐GFP fusion proteins. Parental 293T cells were plated in 96‐well plates (2 × 10^4^ cells/well) and incubated with EVs (∼2 × 10^6^) at 37°C for 3, 6, 12, 18 and 24 h. Cells were then washed three times with PBS and evaluated for EV uptake by measuring GFP fluorescence intensity using a Spark® microplate reader. Untreated 293T cells were used as a blank. Uptake of a given set of marker‐positive EVs was assessed in terms of GFP fluorescence at each time‐point relative to GFP fluorescence at 24 h after EV addition. Four independent experiments were performed for each assay, where each experiment used a different batch of EVs.

### 2.14 Isolation and quantification of miRNA

Prior to isolating miRNA, all batches of EVs were treated with 0.2 μg/mL of RNase A for 30 min at 37°C to remove external RNA. miRNA was isolated using the two‐column PureLink™ miRNA isolation kit (Invitrogen) following manufacturer's instructions. Briefly, EVs or cells were lysed with Trizol™ reagent, followed by the addition of chloroform and centrifugation at 12,000 × *g* for 15 min at 4°C. The aqueous phase was collected, mixed with an equal volume of 100% ethanol, and then loaded onto the first column to retain large RNA. Following centrifugation of the column at 12,000 × *g* for 1 min, the flow‐through was collected, mixed with a 2‐fold volume of 100% ethanol and then loaded onto the second column to retain small RNA. The second column was centrifuged at 12,000 × *g* for 1 min and washed twice with washing buffer provided in the kit. Small RNA was eluted from the second column by the addition of RNase‐free water (50 μL) and centrifugation at 12,000 × *g* for 1 min. miRNA was isolated from equivalent volumes of body fluid samples (100 μL of mouse plasma, 200 μL of human plasma or ascites) by three methods. In the direct lysis method, 600 μL of Trizol™ reagent was directly added to the fluid sample, and miRNA was isolated as described above. In the precipitation method, the fluid sample was diluted by the addition of 300 μL of PBS, incubated with 126 μL of ExoQuick® reagent (System Biosciences) at 4°C for 16 h, and then centrifuged at 1,500 × *g* for 30 min. The pellet was lysed with Trizol™ reagent, and miRNA was isolated as described above. In the CD147 immunocapture method, the fluid sample was diluted by the addition of 800 μL of PBS, and incubated with CD147 antibody‐conjugated magnetic beads at 4°C for 16 h. Thereafter, beads were washed three times with PBS. Trizol™ reagent was directly added to the beads, and miRNA was isolated as described above. Concentration of miRNA in samples was determined by using the Quant‐iT™ microRNA assay kit (Invitrogen) according to manufacturer's instructions. Size distribution of RNA molecules was evaluated by using a 2100 Bioanalyzer™ with a small RNA chip (Agilent) according to manufacturer's instructions.

### 2.15 RT‐qPCR of miRNA

To normalize miRNA content in EVs and body fluids, cel‐miR‐39 spike‐in control (Qiagen) was added to Trizol™ reagent during miRNA isolation. RT‐qPCR of miRNA was performed by using a Taqman^®^ MicroRNA Reverse Transcription kit (Applied Biosystems) and sequence‐specific stem‐loop primers for hsa‐miR‐302a‐3p, hsa‐miR‐302c‐3p, hsa‐miR‐1233‐3p, hsa‐miR‐210‐3p, cel‐miR‐39‐3p, and RNU‐48 (Applied Biosystems). Reactions were performed in a StepOne Plus™ Real‐Time PCR system with 1X Master Mix and 1X probes (TaqMan^®^ microRNA Expression Assay, Applied Biosystems). Relative levels of miRNAs in EVs were calculated by using the comparative CT method (2‐∆∆CT) and the cel‐miR‐39 spike‐in control for normalization. RNU‐48 was used as an endogenous control for normalization of cellular miRNA levels. Absolute copy numbers of miRNAs in Figure 4b and Figure 8b, d and e were calculated from standard curves that were generated using miRNA mimetics.

### 2.16 miRNA profiling

Reverse transcription of miRNA, isolated from body fluids and tumour tissue, was performed by using the miRCURY LNA™ RT kit (Qiagen) with cel‐miR‐39 as a spike‐in control. qPCR was carried out by using the miRCURY LNA™ miRNA cancer focus PCR panel (Qiagen) that contains primer sets for 84 known cancer‐associated miRNAs. Reactions were performed in a StepOne Plus™ Real‐Time PCR system (Applied Biosystem) with miRCURY SYBR® Green Master Mix (Qiagen). Cycling conditions were as follows: 2 min at 95°C, followed by 40 amplification cycles of 10 s at 95°C and 60 s at 56°C, and by the melting curve. Ct values obtained from the different panels were adjusted by an Inter Plate Calibrator. For each case, only those miRNAs that were detected in at least one of the three fluid‐derived samples were considered for further analysis. The expression level of each miRNA was normalized using the ΔCt method (ΔCt = Ct of each miRNA—Ct of Cel‐miR‐39). For each case, correlations between ΔCt values in each fluid‐derived sample and ΔCt values in matching tumour tissue were assessed by Spearman test.

### 2.17 Statistical analysis

Statistical analysis was performed by using GraphPad Prism 9.0 software (GraphPad Software). Normality of data distribution in groups was assessed by Shapiro‐Wilk test. Significance of data in in vitro and in vivo assays was assessed, where indicated, by unpaired two‐tailed Student's *t*‐test for comparison of two groups, or by one‐way or two‐way ANOVA with Bonferroni's corrections for multiple comparisons. Unless otherwise indicated, multiple comparisons were made of unpaired samples. Data represent means ± SD unless otherwise indicated. *P* values of <0.05 were considered significant.

## 3 RESULTS

### 3.1 CD147 and CD98 define subpopulations of EVs that are distinct from tetraspanin^+^ EVs

**FIGURE 1:**
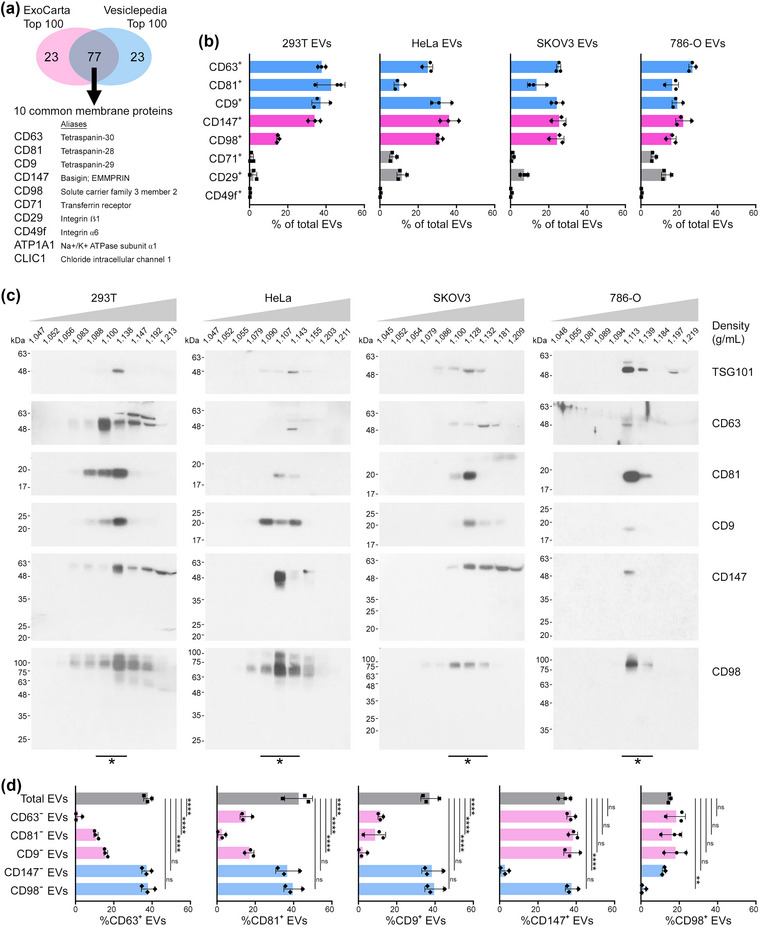
CD147 and CD98 are predominantly expressed in tetraspanin‐negative EVs. (a) Venn diagram of unique and common top 100 EV proteins in the ExoCarta and Vesiclepedia databases. (b) Percentages of EVs derived from 293T, HeLa, SKOV3 and 786‐O cells that express the indicated membrane proteins. Mean ± SD of *n* = 3 independent experiments are shown. Representative flow cytometric analysis of staining is shown in Figure S3B. (c) Fractions of the indicated buoyant densities were isolated by density gradient ultracentrifugation from media conditioned by 293T, HeLa, SKOV3 and 786‐O cells, and assayed for CD63, CD81, CD9, CD147 and CD98 by immunoblot. As a positive control, fractions were assayed for tumour susceptibility gene 101 (TSG101), an exosomal cargo protein. EV‐containing fractions of buoyant densities of 1.09–1.14 g/mL are indicated by asterisks. Immunoblot data was verified in three independent experiments. (d) Batches of total EVs were depleted of EVs that express a given surface marker (Marker A). The remaining pool of EVs (Marker A‐negative subpopulation) and the total EV population were assayed for other surface markers (refer Figure S4A, B). Shown are the percentages of EVs that express either CD63, CD81, CD9, CD147 or CD98 in the total EV population and in the indicated subpopulations of marker‐negative EVs derived from 293T cells. Mean ± SD of *n* = 3 independent experiments are shown. Representative flow cytometric analysis of staining is shown in Figure S5A. ns, not significant, **P < 0.01, ****P < 0.0001, by one‐way ANOVA with Bonferroni's corrections in d.

We initially reviewed the two largest databases of proteins that have been identified in EVs derived from diverse cell types and body fluids (Kalra et al., 2012; Keerthikumar et al., 2016). The 100 most frequently identified EV proteins in the ExoCarta and Vesiclepedia databases contained 77 common proteins of which 10 are membranous (Figure 1a). These include three tetraspanins (CD63, CD81, CD9) and seven other less‐characterized proteins. Expression of the tetraspanins and five other common membrane proteins (CD147, CD98, CD71, CD29, CD49f) was confirmed in human cell lines of diverse origin, namely, 293T (embryonic kidney), HeLa (cervical carcinoma), SKOV3 (ovarian carcinoma, OVCA) and 786‐O (renal cell carcinoma, RCC) (Figure S1). EVs were isolated from media conditioned by these cell lines using a method we previously optimized that includes ultrafiltration to remove soluble non‐EV proteins and particulates, followed by fractionation based on buoyant density (Ko et al., 2019). Purified EVs were visualized by transmission electron microscopy to confirm their intact membranous structure (Figure S2A), and their size distribution was determined by nanoparticle tracking analysis (Figure S2B). The prevalence of surface proteins in EVs was evaluated by flow cytometry using an approach that we applied in a previous study (Ko et al., 2019). Settings were optimized for EV detection by acquiring microbeads of various diameters in the size range of EVs, and then used to detect surface staining of proteins in EVs (Figure S3A). About 8%–47% of EVs contained either CD63, CD81 or CD9 (Figures 1b and S3B). CD147 and CD98 were detected in 19%– 41% and 13%–33% of EVs, respectively, whereas CD71, CD29 and CD49f were detected in only 0.1%–16% of EVs (Figures 1b and S3B). A study of protein topology in EVs has identified that several membrane proteins are displayed in EVs in an orientation that is reverse to that in the plasma membrane, that is ‘inside‐out’ (Cvjetkovic et al., 2016). This raises the possibility that the low detection of CD71, CD29 and CD49f in EVs might stem from masking of antibody epitopes. These markers were therefore excluded from further analysis. To confirm that CD147, CD98 and the three tetraspanins are associated with EVs, all density gradient fractions were evaluated for these markers by immunoblot. Full‐length forms of all five proteins, and not truncated forms corresponding to shed ectodomains, were detected in fractions within the buoyant density range of EVs (Figure 1c).

We next evaluated co‐expression of CD147, CD98 and the three tetraspanins in EVs. Tetraspanins act as membrane‐organizing scaffolds by engaging with one another and other membrane proteins and protrude only 4–5 nm from the membrane (Hemler, 2005; Kitadokoro et al., 2001). Because of steric hindrance, it is difficult to reliably detect these proteins by co‐staining with multiple antibodies. To overcome this limitation, we depleted EVs that express a given surface marker (Marker A), and then determined the proportion of EVs that express another surface marker (Marker B) in the remaining pool of Marker A‐negative EVs (post‐depletion) and in the original total EV population (pre‐depletion) (Figure S4A, B). This approach was initially used to analyse EVs derived from 293T cells. As compared to the total EV population, depletion of EVs that express a given tetraspanin significantly reduced the proportions of EVs that express either of the other two tetraspanins (Figures 1d and S5A). These findings indicate that the majority but not the entirety of tetraspanin^+^ EVs coexpress at least two tetraspanins, and are in keeping with several reports (Han et al., 2021; Mathieu et al., 2021; Tian et al., 2018). By contrast, depletion of either CD147^+^ EVs or CD98^+^ EVs did not significantly reduce the proportions of EVs that are CD63^+^, CD81^+^ or CD9^+^ (Figures 1d and S5A). Conversely, depletion of either CD63^+^ EVs, CD81^+^ EVs or CD9^+^ EVs did not reduce the proportions of EVs that are CD147^+^ or CD98^+^ (Figures 1d and S5A). Similar results were obtained in depletion experiments using EVs derived from HeLa, SKOV3 and 786‐O cells (Figure S5B–E). These findings strongly indicate that CD147 and CD98 are predominantly expressed in tetraspanin‐negative EVs. To confirm these findings, we performed triple depletion of CD63^+^ EVs, CD81^+^ EVs and CD9^+^ EVs, and observed that the remaining pool of tetraspanin‐negative EVs was significantly enriched in EVs that are CD147^+^ or CD98^+^ (Figure S5F). Furthermore, analysis of CD147 expression in CD98‐negative EVs, and of CD98 expression in CD147‐negative EVs, revealed that expression of CD147 and CD98 in EVs is almost mutually exclusive (Figures 1d and S5A–E).

### 3.2 Biogenesis of CD147^+^ and CD98^+^ EVs is distinct from that of tetraspanin^+^ EVs

**FIGURE 2:**
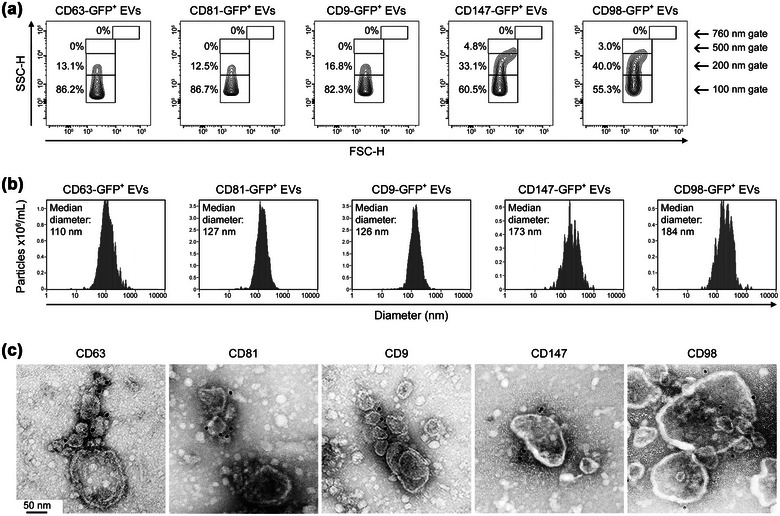
CD147^+^ and CD98^+^ EVs are larger than tetraspanin^+^ EVs. (a, b) Size distribution of EVs derived from 293T cells that express either CD63, CD81, CD9, CD147 or CD98 as GFP‐fusion proteins. In (a), representative forward scatter versus side scatter plots of gated GFP^+^ EVs, with estimates of size distribution based on size marker bead gates (refer Figure S3A). In (b), size distributions of GFP^+^ EVs evaluated by fluorescence nanoparticle tracking analysis. Each plot shows the combined result of 10 replicate measurements. (c) Immunogold labelling of markers in EVs derived from HeLa cells.

Two broad types of EVs released by live cells have been described in terms of their subcellular origin. Exosomes are derived from multivesicular endosomes and range from 30 to 150 nm in diameter, whereas microvesicles (ectosomes) form through outward budding of the plasma membrane and range from 100 nm to 1 μm in diameter (Maas et al., 2017; Mathieu et al., 2019; Xu et al., 2018). To further investigate the possibility that CD147^+^ EVs and CD98^+^ EVs are distinct from tetraspanin^+^ EVs, we evaluated the sizes of these EVs. A limitation to determining the size of EVs that express a given surface marker is that binding of antibody to the marker alters EV size, and it is difficult to detach the antibody without damaging the integrity of EVs. To overcome this limitation, we generated 293T cell lines that express either CD63, CD81, CD9, CD147 or CD98 as GFP‐fusion proteins, and evaluated the size of GFP^+^ EVs derived from each of these lines by flow cytometry and by fluorescence nanoparticle tracking analysis. These two independent analyses showed that CD147^+^ EVs and CD98^+^ EVs are larger than tetraspanin^+^ EVs (Figure 2a, b). To confirm these findings, we performed immunogold labelling of endogenous surface proteins in EVs. Tetraspanins were mostly detected in smaller EVs (Figure 2c), in keeping with reports that these proteins are contained in exosomes though not exclusively (Escola et al., 1998; Kowal et al., 2016; Mathieu et al., 2021). By contrast, CD147 and CD98 were mostly detected in larger EVs (Figure 2c).

**FIGURE 3:**
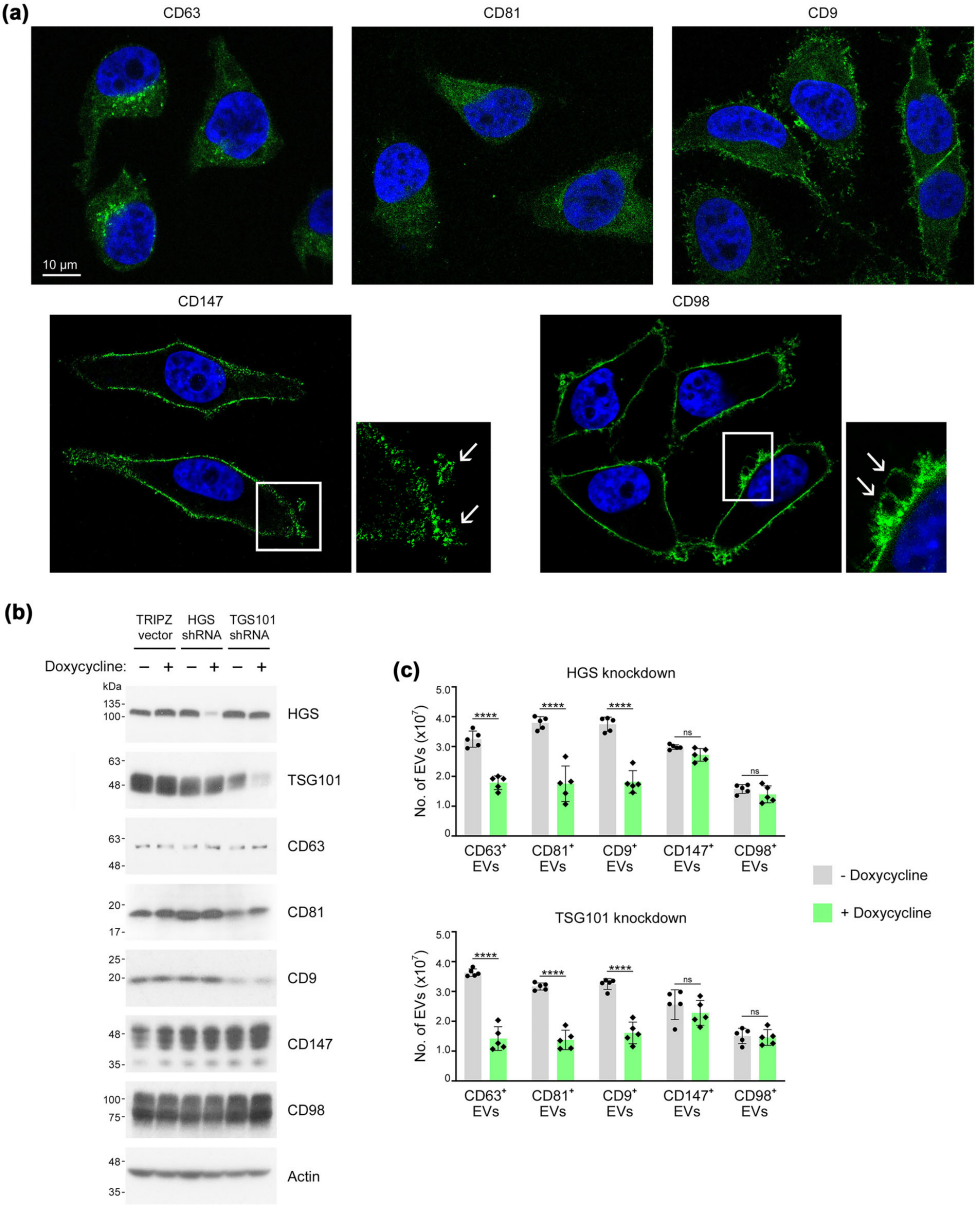
Biogenesis of CD147^+^ and CD98^+^ EVs is distinct from that of tetraspanin^+^ EVs. (a) Subcellular localization of CD63, CD81, CD9, CD147 and CD98 detected by immunofluorescence staining in HeLa cells and visualized by confocal microscopy. Nuclei were visualized by staining with DAPI. Enlarged insets show the presence of CD147 and CD98 in regions of the plasma membrane that bleb outwardly or have pinched off (denoted by arrows). (b, c) 293T cells that express the tetracycline repressor protein were stably transfected with tetracycline‐regulated HGS shRNA or TSG101 shRNA, or with TRIPZ vector. In (b), levels of HGS, TSG101 and the indicated surface markers detected by immunoblot in equivalent amounts of cell lysates (20 μg) of untreated and doxycycline‐treated cells. In (c), numbers of CD63+, CD81+, CD9+, CD147+ and CD98+ EVs produced by equivalent numbers of 293T cells (∼5 × 10^6^) without and following doxycycline‐induced knockdown of HGS and TSG101. Mean ± SD of *n* = 5 independent experiments are shown. ns, not significant, ****P < 0.0001, by unpaired two‐tailed Student's *t*‐test in c.

Confocal microscopy revealed that CD147 and CD98 predominantly localize to the plasma membrane (Figure 3a). By contrast, CD63 and CD81 mostly localize to the cytoplasm and CD9 is both cytoplasmic and membranous (Figure 3a). These differences in subcellular localization implicated divergence in the biogenesis of CD147^+^ and CD98^+^ EVs and of tetraspanin^+^ EVs. The most characterized pathway of exosome biogenesis is orchestrated by the ESCRT machinery that comprises four multi‐subunit complexes (ESCRT‐0, ‐I, ‐II, ‐III) and several accessory components (Henne et al., 2011). Hepatocyte growth factor‐regulated tyrosine kinase substrate (HGS, also known as HRS) and tumour susceptibility gene 101 (TSG101) are core components of ESCRT‐0 and ESCRT‐I, respectively (Henne et al., 2011), and knockdown of these components inhibits exosome secretion (Colombo et al., 2013). Expression levels of CD147, CD98 and the three tetraspanins were not altered by knockdown of HGS or TSG101 (Figure 3b). Notably, knockdown of either HGS or TSG101 significantly inhibited secretion of tetraspanin^+^ EVs, but did not affect secretion of CD147^+^ EVs or CD98^+^ EVs (Figure 3c). These findings indicate that the vast majority of CD147^+^ EVs and CD98^+^ EVs are not exosomes. Visualization of cells at high magnification revealed the presence of CD147 and CD98 in regions of the plasma membrane that bud outwardly and pinch off (Figure 3a). Taken together, these findings support the notion that CD147^+^ EVs and CD98^+^ EVs most likely are microvesicles and represent subpopulations of EVs that are distinct from tetraspanin^+^ EVs.

### 3.3 CD147^+^ EVs transport biologically active miRNAs into recipient cells

**FIGURE 4:**
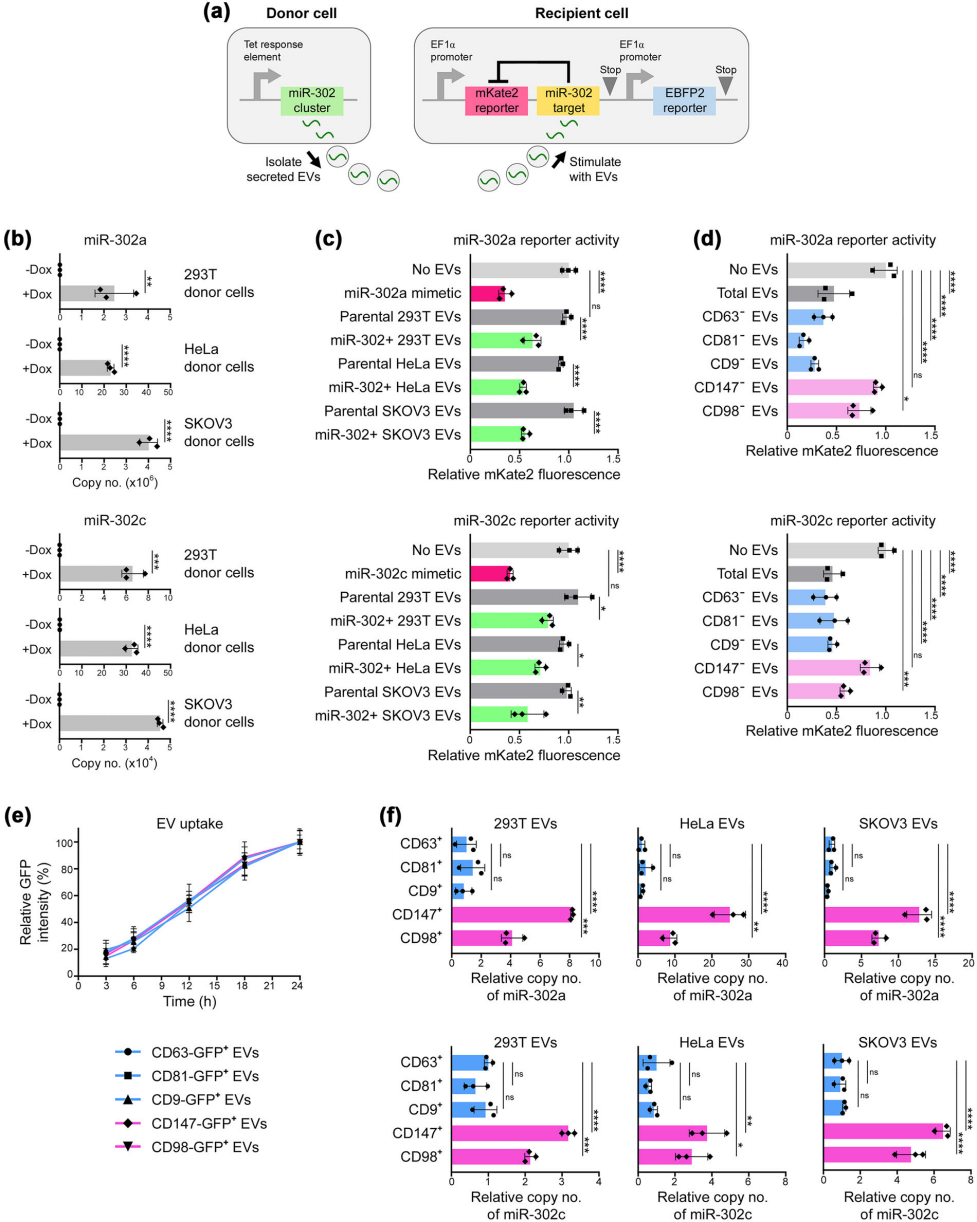
CD147^+^ EVs transport biologically active miRNAs into recipient cells. (a) Schematic of assay system for EV‐mediated transfer of miR‐302. (b) Copy numbers of miR‐302a and miR‐302c in untreated and doxycycline‐treated 293T, HeLa and SKOV3 donor cells. Mean ± SD of *n* = 3 independent experiments are shown. (c, d) 293T recipient cells that express mKate2 with either a 3′ miR‐302a or miR‐302c target sequence were stimulated with EVs. mKate2 fluorescence was measured at 48 h thereafter and expressed relative to fluorescence intensity in unstimulated recipient cells. In (c), recipient cells were stimulated with comparable numbers of EVs (∼2 × 10^6^) derived from parental and miR‐302‐overexpressing donor cells. As positive controls, recipient cells were transfected with miR‐302a or miR‐302c mimetics. In (d), comparable numbers of EVs (∼2 × 10^6^) derived from miR‐302‐overexpressing HeLa donor cells were depleted of EVs that express either CD63, CD81, CD9, CD147 or CD98 or left undepleted, and thereafter used to stimulate recipient cells. Mean ± SD of *n* = 3 independent experiments are shown in c and d. (e) Uptake of EVs was evaluated in parental 293T cells following addition of comparable numbers of EVs (∼2 × 10^6^) derived from 293T cells that stably express either CD63, CD81, CD9, CD147 or CD98 as GFP‐fusion proteins. Uptake of a given set of EVs is expressed in terms of GFP fluorescence intensity at each indicated time‐point relative to GFP fluorescence intensity at 24 h after EV addition. Mean ± SD of *n* = 4 independent experiments are shown. (f) Comparable numbers of CD63^+^, CD81^+^, CD9^+^, CD147^+^ and CD98^+^ donor cell‐derived EVs (∼2 × 10^7^) were evaluated for relative copy numbers of miR‐302a and miR‐302c. Copy number of cel‐miR‐39 spike‐in control was used for normalization. Mean ± SD of *n* = 3 independent experiments are shown. ns, not significant, *P <  0.05, **P <  0.01, ***P <  0.001, ****P <  0.0001 by unpaired two‐tailed Student's *t*‐test in b; by one‐way ANOVA with Bonferroni's corrections in c, d and f.

Of the EV cargo, miRNAs have attracted substantial interest but several elegant studies have disputed the content and significance of miRNAs in EVs (Albanese et al., 2021; Arroyo et al., 2011; Chevillet et al., 2014; Zhang et al., 2021). To investigate the possibility that miRNAs are enriched in only a subpopulation of EVs, we firstly established an assay system to evaluate the export of miRNA by EVs secreted by donor cells and the import of bioactive miRNA by EVs into recipient cells (Figure 4a). To ensure that miRNA bioactivity in recipient cells can be attributed to EV‐mediated miRNA transfer, we chose the miR‐302 cluster as a test miRNA because it is not endogenously expressed in mature cells (Suh et al., 2004). We generated donor cell lines in which miR‐302 expression is induced by doxycycline (Figure 4b), and recipient cell lines that express a dual‐reporter cassette containing a miR‐302 target sequence (Gam et al., 2018) (Figure 4a). Bioactivity of miR‐302, that is transported in donor cell‐derived EVs and taken up by recipient cells, was assayed by measuring mKate2 fluorescence (Figure 4a). Robustness of the system was confirmed by the inhibition of mKate2 fluorescence in recipient cells following stimulation with EVs derived from miR‐302‐expressing donor cells (Figure 4c). Donor cell‐derived EVs were then depleted of EVs that express a given marker, and the remaining marker‐negative EVs were used to stimulate recipient cells. As compared to unstimulated cells, miR‐302 bioactivity did not decrease following stimulation with CD147‐negative EVs, but was decreased by 27%–41% following stimulation with CD98‐negative EVs (Figure 4d). Stimulation with tetraspanin‐negative EVs more greatly decreased miR‐302 bioactivity (by 53%–83%) and was as effective as stimulation with total (non‐depleted) EVs (Figure 4d). No significant differences were found in the rates of uptake of tetraspanin^+^ EVs, CD147^+^ EVs and CD98^+^ EVs by recipient cells (Figure 4e). These results indicate that the vast majority of EV‐associated miR‐302 is contained in tetraspanin‐negative EVs. Consistent with our findings that CD147 and CD98 are predominantly expressed in tetraspanin‐negative EVs, copy numbers of miR‐302 were significantly higher in CD98^+^ EVs than in tetraspanin^+^ EVs, and even higher in CD147^+^ EVs (Figure 4f).

### 3.4 CD147^+^ EVs are enriched in miRNA through the interaction of CD147 with hnRNP A2/B1

**FIGURE 5:**
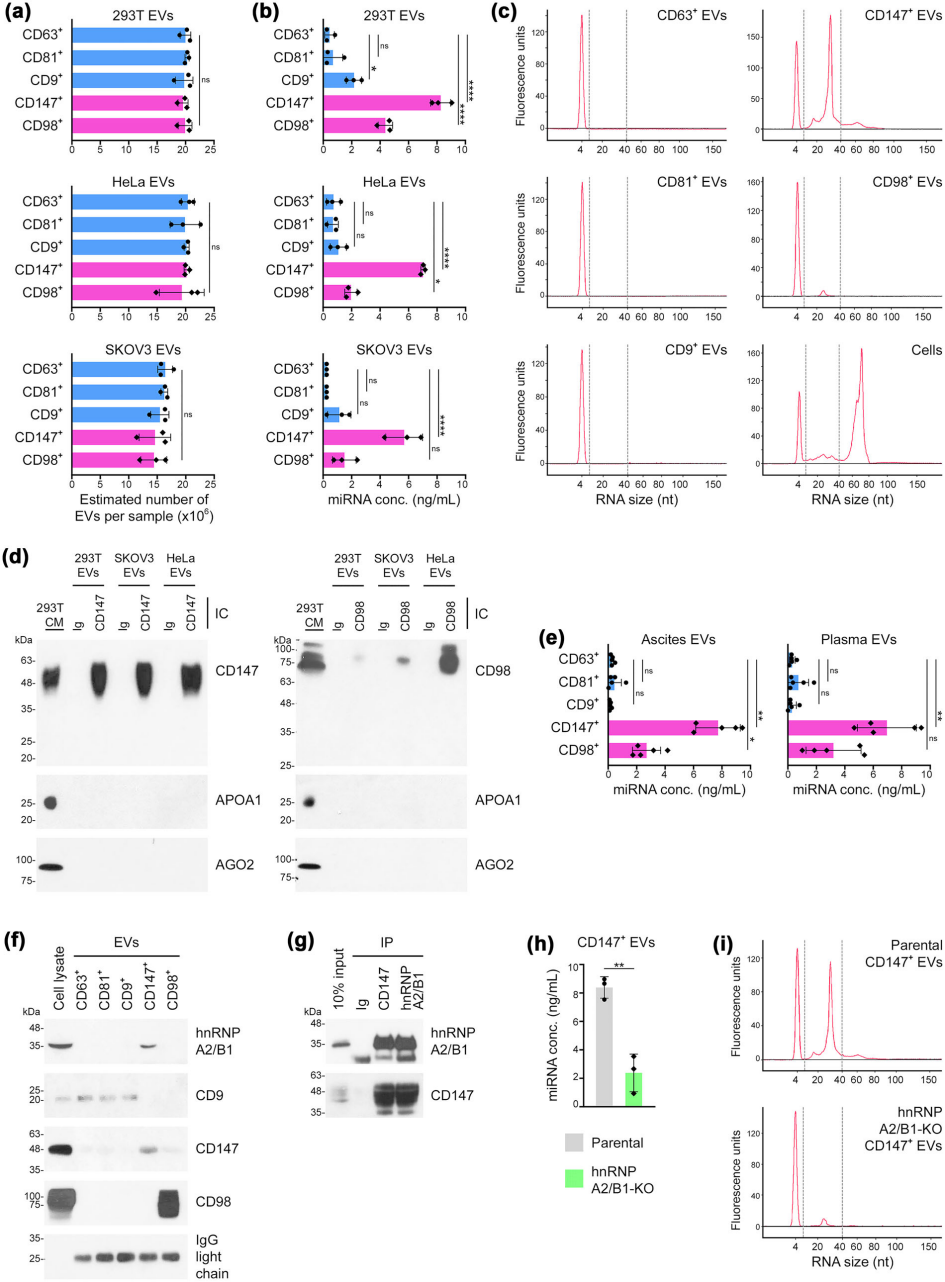
CD147^+^ EVs are enriched in miRNA through the interaction of CD147 with hnRNP A2/B1. (a, b) Comparable numbers of CD63^+^, CD81^+^, CD9^+^, CD147^+^ and CD98^+^ EVs derived from 293T, HeLa and SKOV3 cells were evaluated for miRNA content. In (a), numbers of marker‐positive EVs, calculated from the difference between EV counts in the initial input of total EVs and in the supernatant following immunocapture of EVs that express a given marker. In (b), total miRNA concentrations. Mean ± SD of *n* = 3 independent experiments are shown in a and b. (c) Agilent 2100 Bioanalyzer™ electropherogram profiles of small RNA isolated from HeLa cells and from comparable numbers of marker‐positive HeLa cell‐derived EVs (∼2 × 10^7^). Small RNAs detected between the two dotted lines were considered as miRNAs. The peak at 4 nt corresponds to the loading control. (d) To confirm that CD147^+^ and CD98^+^ EVs are not contaminated with HDL or AGO2 complexes, total EVs were isolated from conditioned media of 293T, SKOV3 and HeLa cells, followed by immunocapture (IC) using antibodies to CD147, CD98 or Ig isotype control. Lysates of immunocaptured material were assayed by immunoblot for APOA1, the major protein constituent of HDL, and for AGO2. Conditioned media (CM) of 293T cells was included as a positive control for APOA1 and AGO2. (e) Total miRNA concentration in marker‐positive EVs isolated from ascites of OVCA patients (*n* = 5) and from plasma of patients with OVCA (*n* = 2) or RCC (*n* = 3). (f) Immunoblot of hnRNP A2/B1 in marker‐positive EVs derived from 293T cells. (g) Interaction of CD147 with hnRNP A2/B1 in 293T cells detected by immunoprecipitation (IP). (h, i) Comparable numbers of CD147^+^ EVs derived from parental and hnRNP A2/B1‐KO 293T cells were evaluated for total miRNA concentration (h) and small RNA content (i), as described in b and c. Mean ± SD of *n* = 3 independent experiments are shown in h. ns, not significant, *P < 0.05, **P < 0.01, ****P < 0.0001 by one‐way ANOVA with Bonferroni's corrections in a, b and e, where paired samples were analysed in e; by unpaired two‐tailed Student's *t*‐test in h.

In subsequent studies, we isolated tetraspanin^+^ EVs, CD147^+^ EVs and CD98^+^ EVs that are secreted by 293T, HeLa and SKOV3 cells, and measured the total miRNA content in comparable numbers of EVs of each subpopulation (Figure 5a). Tetraspanin^+^ EVs had the lowest miRNA content (Figure 5b). As compared to tetraspanin^+^ EVs, the total miRNA content was significantly though modestly higher (2–4‐fold) in CD98^+^ EVs derived from two of the cell lines, and substantially higher (8–17‐fold) in CD147^+^ EVs derived from all three cell lines (Figure 5b). Enrichment of miRNA in CD147^+^ EVs was confirmed by analysing the small RNA content in each subpopulation of EVs using an Agilent 2100 Bioanalyzer™ (Figures 5c and S6). To eliminate the possibility that miRNAs are associated with non‐EV components, purified EVs in all experiments were treated with RNase prior to miRNA analysis. Because non‐vesicular extracellular miRNAs form complexes with high‐density lipoprotein (HDL) (Vickers et al., 2011) and with Argonaute 2 (AGO2) (Arroyo et al., 2011), we confirmed that apolipoprotein A1 (APOA1), the major protein constituent of HDL, and AGO2 are not detectable in CD147^+^ EVs or CD98^+^ EVs (Figure 5d). To confirm our findings in clinical samples, we analysed EVs that were isolated from OVCA patient ascites and from plasma of patients with OVCA and RCC, and similarly found significant miRNA enrichment in CD147^+^ EVs (i.e. 9–26‐fold higher than tetraspanin^+^ EVs) (Figure 5e).

Several RNA‐binding proteins control sorting of miRNA into EVs and have been detected in EVs (Lee et al., 2019; Santangelo et al., 2016; Temoche‐Diaz et al., 2019; Villarroya‐Beltri et al., 2013). One of these RNA‐binding proteins, hnRNP A2/B1, was detected in CD147^+^ EVs but not in tetraspanin^+^ EVs or CD98^+^ EVs (Figure 5f). To investigate the significance of hnRNP A2/B1 in CD147^+^ EVs, we used 293T cells in which the *HNRNPA2B1* gene was deleted by CRISPR/Cas9 gene editing (hnRNP A2/B1‐KO). Knockout of hnRNP A2/B1 did not alter cellular expression levels of CD147, CD98 and tetraspanins (Figure S7A), or the expression of these surface markers in EVs (Figure S7B, C). Notably, immunoprecipitation assays revealed that CD147 interacts with hnRNP A2/B1 (Figure 5g). Furthermore, the total miRNA content in CD147^+^ EVs derived from hnRNP A2/B1‐KO cells was significantly lower than the total miRNA content in CD147^+^ EVs derived from parental 293T cells (Figure 5h, i). These findings implicate that the selective enrichment of miRNA in CD147^+^ EVs occurs through the binding of hnRNP A2/B1 to miRNA and its interaction with CD147.

### 3.5 CD147 is a candidate surface marker of cancer cell‐derived EVs

Almost all types of cells release EVs, and there are no well‐defined surface markers that can distinguish EVs that are released by cancer cells into body fluids. CD147 and CD98 are overexpressed in a variety of solid tumours, but are also expressed in endothelial cells, leukocytes and some types of normal epithelium such as renal tubular epithelium (Cantor & Ginsburg 2012; Kosugi et al., 2015; Liao & Cantor, 2016; Xin et al., 2016; Xiong et al., 2014). To determine whether normal cells secrete CD147^+^ and CD98^+^ EVs, we evaluated renal proximal tubule epithelial cells (RPTEC) which are widely thought to be the cellular origin of clear cell RCC. Normal RPTEC expressed CD147 and CD98 at significantly lower levels than 786‐O RCC cells (Figure S8A), and secreted significantly fewer CD147^+^ and CD98^+^ EVs than equivalent numbers of 786‐O cells (Figure S8B). Similarly, normal endothelial cells expressed CD147 and CD98 at lower levels than 786‐O cells (Figure S8A), and secreted fewer CD147^+^ and CD98^+^ EVs (Figure S8B). By contrast, the numbers of tetraspanin^+^ EVs secreted by RPTEC and endothelial cells were not significantly different from numbers of tetraspanin^+^ EVs secreted by 786‐O cells (Figure S8B). These findings raise the possibility that CD147 and CD98 might enable identification of cancer cell‐derived EVs, and that CD147^+^ miRNA‐enriched EVs are predominantly secreted by cancer cells.

**FIGURE 6:**
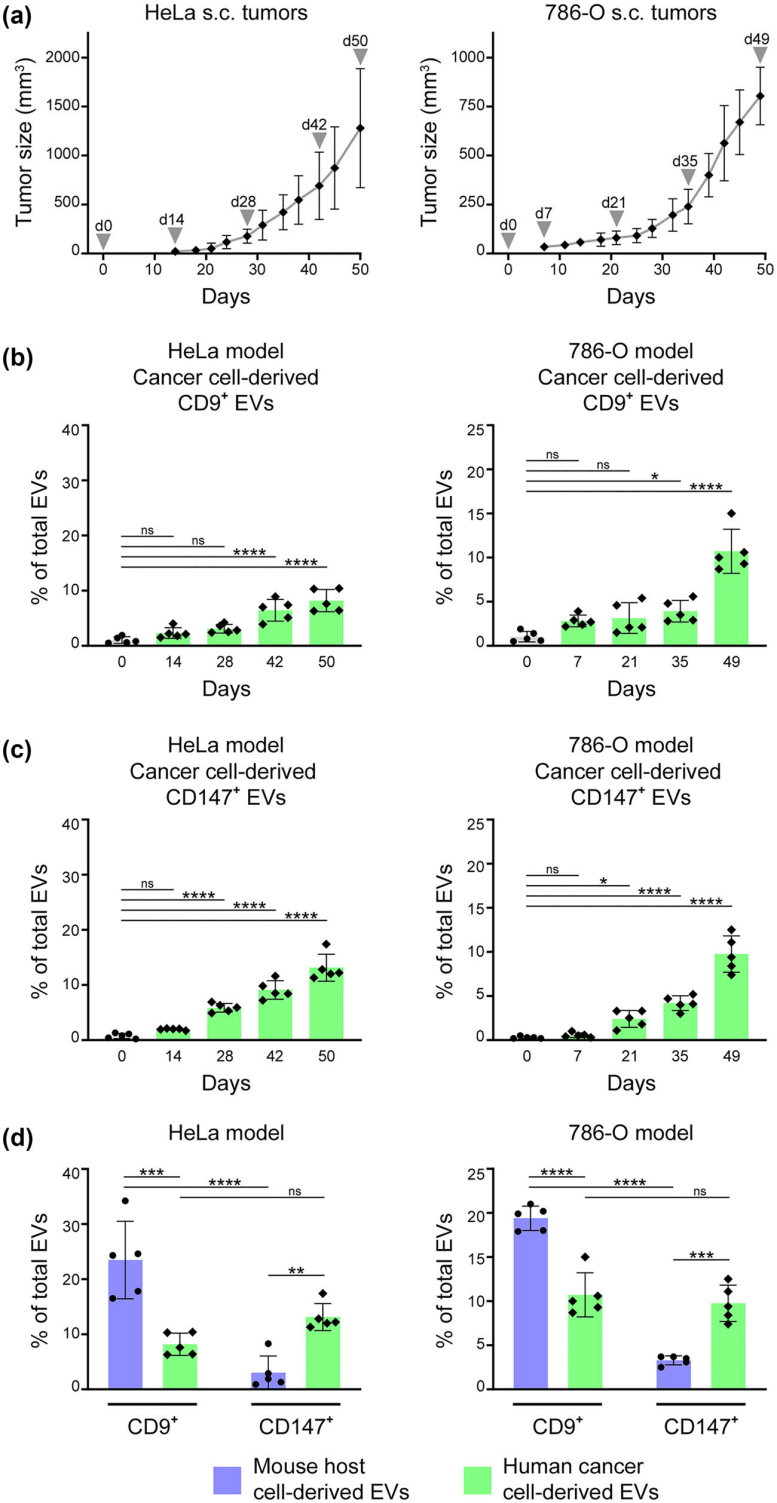
CD147^+^ EVs predominantly derive from cancer cells. Nude mice were inoculated s.c. with HeLa cells or with 786‐O cells. (a) Growth of tumours. Peripheral blood was collected at the indicated time‐points. (b‐d) Plasma EVs that derive from human cancer cells and from non‐cancerous mouse host cells were distinguished by staining with antibodies specific to human and mouse markers. Shown are percentages of CD9^+^ EVs (b) and CD147^+^ EVs (c) that derive from human cancer cells at each time‐point, and percentages of CD9^+^ EVs and CD147^+^ EVs that derive from human cancer cells (green columns) and from mouse host cells (purple columns) at the terminal time‐point (d). Mean ± SD of results in *n* = 5 mice are shown in a‐d. ns, not significant, *P < 0.05, **P < 0.01, ***P < 0.001, ****P < 0.0001 by one‐way ANOVA with Bonferroni's corrections in b and c; by two‐way ANOVA with Bonferroni's corrections for paired samples in d.

To identify the cell‐of‐origin of CD147^+^ miRNA‐enriched EVs in body fluids, we analysed CD147^+^ EVs in longitudinally collected plasma samples of mice bearing human tumour xenografts. For comparison, we analysed mouse plasma EVs that express the tetraspanin CD9. Cancer cell‐derived EVs were distinguished from non‐cancerous host cell‐derived EVs by using antibodies that are specific to human and mouse surface markers, respectively. Validation of species‐specificity of antibodies is shown in Figure S9A. In mice with HeLa xenografts, the prevalence of CD9^+^ EVs and CD147^+^ EVs that are cancer cell‐derived progressively increased with tumour size (Figure 6a‐c). Notably, the significant increase in prevalence of CD147^+^ EVs was detected substantially earlier than for CD9^+^ EVs, that is at day 28 for CD147^+^ EVs when mean tumour size is 176 mm^3^ (Figure 6c) versus day 42 for CD9^+^ EVs when mean tumour size is 691 mm^3^ (Figure 6b). Similar results were obtained in mice with 786‐O xenografts (Figure 6a‐c). Furthermore, comparative analysis of EVs based on their cellular origin revealed that CD147^+^ EVs predominantly derive from cancer cells in both the HeLa and 786‐O models (Figures 6d and S9B). By contrast, the majority of CD9^+^ EVs were found to derive from non‐cancerous host cells (Figures 6d and S9B). These findings demonstrate that CD147^+^ EVs are predominantly released by cancer cells and from an early stage, and raise the possibility that increased levels of CD147^+^ EVs could be a useful indicator of early‐stage and/or low‐volume malignancy.

### 3.6 CD147^+^ EVs increase in prevalence in cancer patients from early stages of disease

**FIGURE 7:**
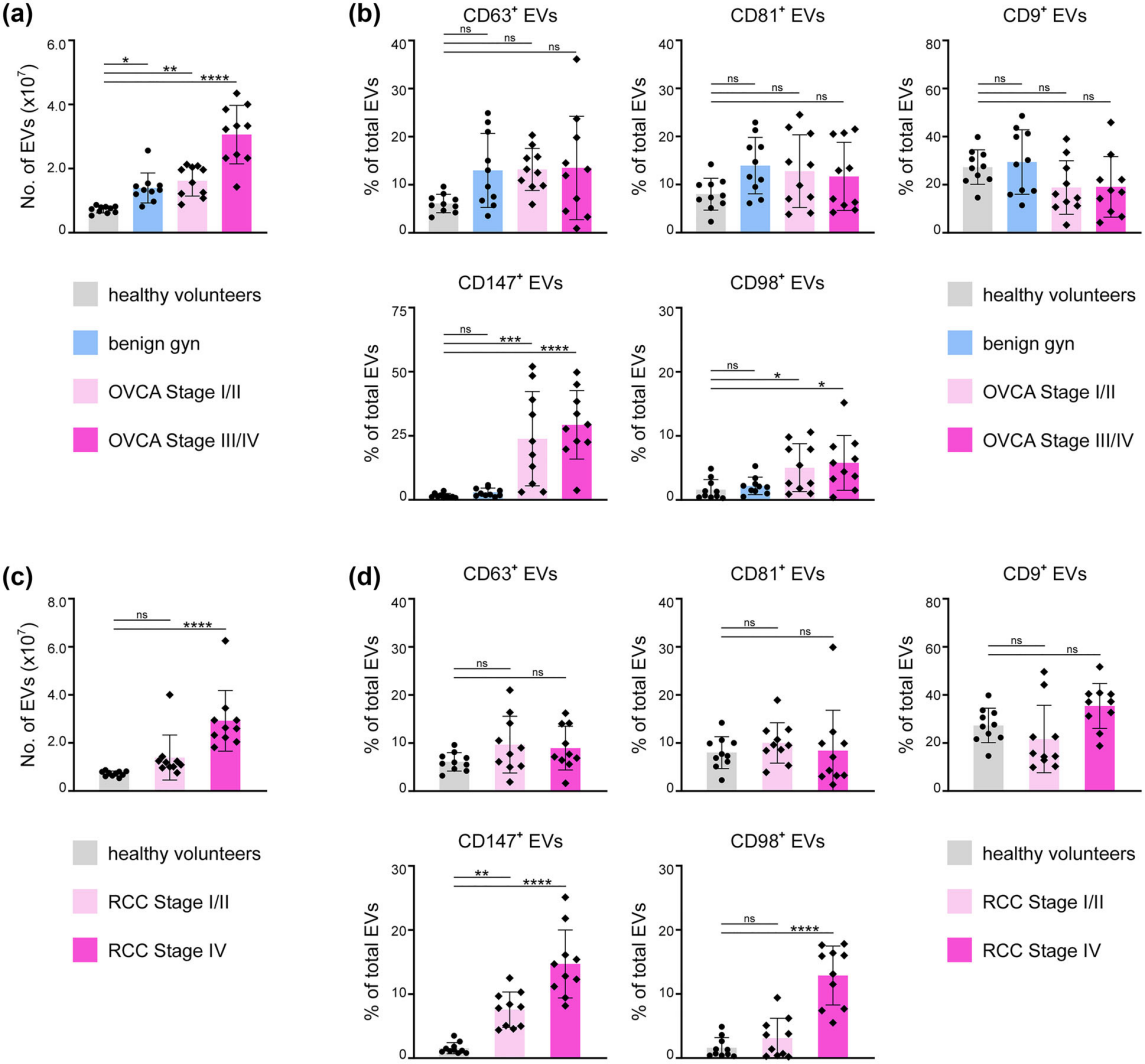
CD147^+^ EVs increase in prevalence in cancer patients from early stages of disease. Analysis of EVs isolated from equivalent volumes (200 μL) of plasma of (a, b) healthy adult volunteers and patients with either benign gynaecologic (gyn) conditions, Stage I/II OVCA or Stage III/IV OVCA, and (c, d) healthy adult volunteers and patients with either Stage I/II RCC or Stage IV RCC. Total numbers of EVs in plasma samples are shown in a and c. Percentages of EVs that express either CD63, CD81, CD9, CD147 or CD98 are shown in b and d. Representative flow cytometric analyses of staining are shown in Figures S10B and S11. Data of healthy volunteers is duplicated in a and c, and in b and d. Mean ± SD of *n* = 10 cases per group are shown in a‐d. ns, not significant, *P < 0.05, **P < 0.01, ***P < 0.001, ****P < 0.0001 by one‐way ANOVA with Bonferroni's corrections in a‐d.

There is substantial evidence that EVs mediate cancer progression (Clement et al., 2020; Czystowska‐Kuzmicz et al., 2019; Ko et al., 2019; Xu et al., 2018; Zhou et al., 2014), but the heterogeneity of EVs in cancer is poorly understood. To gain insight, we analysed EVs in plasma samples of healthy adult volunteers and patients with either benign gynaecologic conditions or OVCA. Clinicopathologic features of cases are described in Table S2. As compared to healthy individuals, total numbers of EVs were elevated in patients with benign conditions and with early‐stage OVCA, and more so in patients with advanced‐stage OVCA (Figure 7a). Plasma levels of CA125, the most widely used OVCA biomarker, were elevated in OVCA patients, but alone could not differentiate patients with benign conditions and those with early‐stage OVCA (Figure S10A). The prevalence of tetraspanin^+^ EVs did not significantly differ between healthy individuals and patients with either benign conditions or OVCA (Figures 7b and S10B). Similar trends were found in plasma of patients with RCC. Total numbers of EVs were significantly elevated in patients with advanced‐stage RCC (Figure 7c), and there was no significant difference in the prevalence of tetraspanin^+^ EVs between healthy individuals and RCC patients irrespective of disease stage (Figures 7d and S11).

In contrast to tetraspanin^+^ EVs, CD147^+^ EVs and CD98^+^ EVs each constituted only a small fraction (∼1.6%) of the total EV population in healthy individuals (Figure 7b). The prevalence of CD98^+^ EVs was modestly (3‐fold) higher in patients with early‐stage and advanced‐stage OVCA (Figures 7b and S10B), and was 8‐fold higher in patients with advanced‐stage RCC but not significantly increased in those with early‐stage RCC (Figures 7d and S11). By contrast, the prevalence of CD147^+^ EVs was nearly 20‐fold higher in patients with early‐stage and advanced‐stage OVCA, and effectively differentiated OVCA patients, patients with benign conditions, and healthy individuals (Figures 7b and S10B). These findings raise the possibility that assaying CD147^+^ EVs could be more efficacious than CA125 for OVCA detection. Similarly, increased prevalence of CD147^+^ EVs was detected in RCC patients with early‐stage disease as well as those with advanced‐stage disease (Figures 7d and S11).

### 3.7 CD147 immunocapture enhances detection of cancer‐derived circulating miRNAs

**FIGURE 8:**
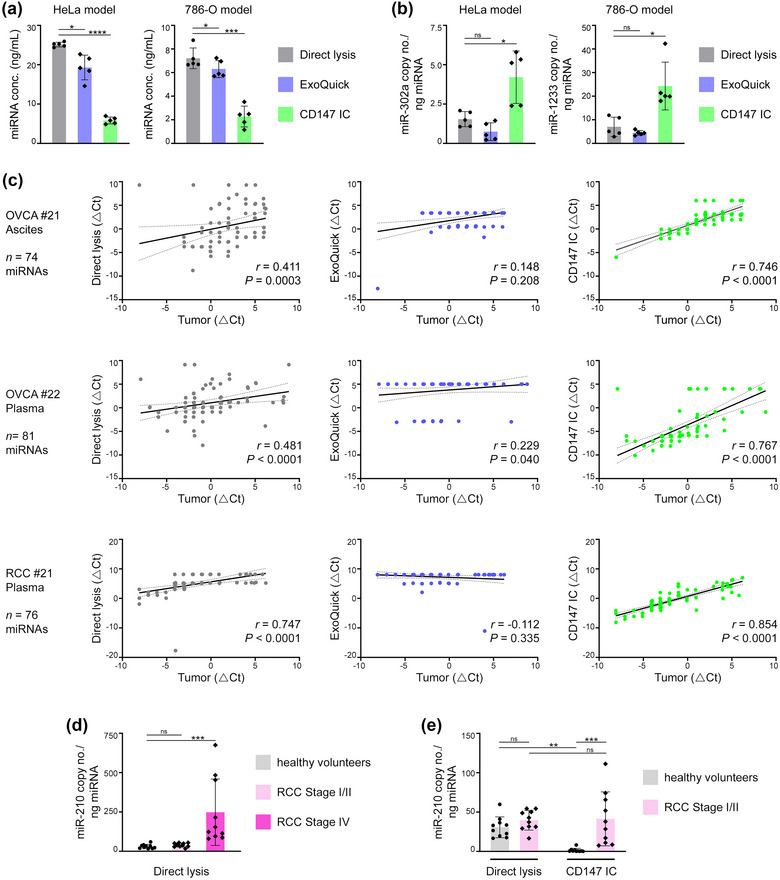
CD147 immunocapture enhances detection of cancer‐derived circulating miRNAs. (a, b) Plasma was collected from mice with tumours derived from miR‐302‐expressing HeLa cells (at Day 50) or from parental 786‐O cells (at Day 49) (refer Figure 6a). miRNA was isolated from equivalent volumes of plasma (100 μL) either by direct lysis of whole plasma, precipitation using ExoQuick® reagent, or immunocapture (IC) with CD147 antibody. In (a), concentrations of total miRNA isolated by each of the three methods. In (b), copy numbers of miR‐302a (HeLa models) and miR‐1233 (786‐O models) detected in equivalent amounts of total miRNA. Mean ± SD of *n* = 5 independent samples are shown in a and b. (c) miRNA was isolated by each of the three methods from equivalent volumes of body fluids (ascites or plasma, 200 μL) of patients with OVCA or RCC. Expression levels of 84 cancer‐associated miRNAs in each of the three fluid‐derived samples of each case were evaluated by using a miRCURY LNA™ miRNA cancer focus PCR panel (Qiagen). For each case, only those miRNAs that were detected in at least one fluid‐derived sample were considered for correlation analysis. Shown are Spearman rank correlations between levels of miRNAs in each fluid‐derived sample and levels of miRNAs in matching tumour tissue of each case. Dotted lines indicate 95% confidence intervals. The number of miRNAs analysed for each case is indicated. (d, e) Copy numbers of miR‐210 detected in plasma of healthy volunteers and patients with either Stage I/II or Stage IV RCC where miRNA was isolated by direct lysis (d), and in the same plasma samples of healthy volunteers and patients with Stage I/II RCC where miRNA was isolated by direct lysis or CD147 immunocapture (e). Data of direct lysis samples is duplicated in d and e. Mean ± SD of *n* = 10 cases per group are shown. ns, not significant, *P  <  0.05, **P < 0.01, ***P  <  0.001, ****P  <  0.0001 by one‐way ANOVA with Bonferroni's corrections in a, b and d, where paired samples were analysed in a and b; by two‐way ANOVA with Bonferroni's corrections in e.

Fractionation based on buoyant density has been widely regarded as the gold standard for isolating EVs at high purity (Théry et al., 2018), and was used to isolate all EVs in this study in conjunction with ultrafiltration. However, this isolation method is labour‐intensive and is impracticable for a clinical laboratory setting. Clinical studies of EV‐associated miRNAs have mostly used other methods to isolate EVs from body fluids rapidly but with less purity, such as precipitation with polymer‐based reagents (e.g. ExoQuick®) (Hannafon et al., 2016; Shin et al., 2021; Wang et al., 2022). EVs in body fluids have also been captured by using antibodies to tetraspanins (Campos‐Silva et al., 2019; Duijvesz et al., 2015; Logozzi et al., 2009). Our findings that CD147 is a candidate surface marker of cancer cell‐derived EVs and that miRNAs are enriched in CD147^+^ EVs raise the possibility that CD147 immunocapture could increase detection of cancer‐derived circulating miRNAs. To investigate this possibility, plasma samples of tumour‐bearing mice were divided into equivalent aliquots from which miRNA was isolated either by direct lysis of whole plasma, precipitation using ExoQuick® reagent, or immunocapture with CD147 antibody. Two groups of xenograft models were evaluated (HeLa, 786‐O). In both groups, the direct lysis and precipitation methods yielded 3–4‐fold more total miRNA than CD147 immunocapture (Figure 8a). To evaluate cancer cell‐specific miRNAs, we assayed miR‐302 in the HeLa model because tumours were established from miR‐302‐overexpressing HeLa cells and miR‐302 is not expressed in normal mature cells (Suh et al., 2004). In the 786‐O model, miR‐1233 was assayed because this miRNA is endogenously expressed in 786‐O cells (Dias et al., 2017) and has no mouse ortholog. Copy numbers of miR‐302a and miR‐1233 were 3–5‐fold higher in miRNA samples that were isolated by CD147 immunocapture than by the other two methods from plasma of mice with HeLa and 786‐O tumours, respectively (Figure 8b). These findings demonstrate that isolating circulating miRNAs by CD147 immunocapture increases the sensitivity of detecting cancer cell‐specific miRNAs.

We next investigated whether miRNAs that are isolated from body fluids of cancer patients by CD147 immunocapture reflect the miRNA expression patterns of tumour tissues. Firstly, miRNA was isolated either by direct lysis, precipitation using ExoQuick® reagent or CD147 immunocapture from equivalent volumes of ascites of OVCA patients. For each case, expression levels of 84 cancer‐associated miRNAs were assessed in each of the three fluid‐derived miRNA samples, and then evaluated for correlations with expression levels of these miRNAs in matching tumour tissue. For all of the three cases analysed, the strongest correlation with the miRNA expression levels in the tumour was obtained with the fluid‐derived miRNA sample that was isolated by CD147 immunocapture (Figures 8c and S12). Similar results were obtained using plasma‐derived samples and matching tumour tissues of patients with either OVCA or RCC (Figure 8c). These findings indicate that circulating miRNAs that are isolated by CD147 immunocapture more closely reflect the tumour miRNA signature than circulating miRNAs that are isolated by conventional methods.

Several independent reports have shown that miR‐210 is overexpressed in RCC and can be detected in the circulation of RCC patients (Dias et al., 2017; Iwamoto et al., 2014; Zhao et al., 2013). To investigate the possibility that isolating circulating miRNAs by CD147 immunocapture might improve the diagnostic performance of a cancer‐associated miRNA, we assayed levels of miR‐210 in plasma samples of the same cohorts of healthy individuals and RCC patients as in Figure 7c and d When circulating miRNA was isolated by direct lysis, a significant difference in miR‐210 copy numbers was detected between healthy individuals and patients with advanced‐stage RCC but not between healthy individuals and patients with early‐stage RCC (Figure 8d). By contrast, a significant difference in miR‐210 copy numbers was detected between healthy individuals and patients with early‐stage RCC when circulating miRNA was isolated by CD147 immunocapture (Figure 8e). Collectively, our findings indicate that CD147 immunocapture could be more effective than conventional methods for isolating cancer‐derived circulating miRNA for liquid biopsy.

## 4 DISCUSSION

The discovery that EVs convey biological information has reshaped our understanding of intercellular communication in homeostasis and disease. However, differentiating EVs in terms of their origin and content within a highly diverse population in body fluids has remained challenging. Several studies have interrogated the biochemical and biophysical heterogeneity among tetraspanin^+^ EVs released by cultured cells (Kowal et al., 2016; Mathieu et al., 2021; Temoche‐Diaz et al., 2019), but the characteristics of tetraspanin‐negative EVs are poorly defined. Here we report that CD147 and CD98 define subpopulations of EVs that are distinct from tetraspanin^+^ EVs. Although we cannot definitively delineate the subcellular origin of CD147^+^ EVs and CD98^+^ EVs, these EVs most likely represent microvesicles as CD147 and CD98 predominantly localize to the plasma membrane and notably in regions that bud outwardly and pinch off. In addition, CD147^+^ EVs and CD98^+^ EVs are produced in an ECSRT‐independent manner and are larger in size than tetraspanin^+^ EVs. We cannot rule out the possibility that CD147, CD98 and tetraspanins might be co‐expressed in a subpopulation of EVs. CD9 has been detected in microvesicles (Mathieu et al., 2021), and CD9^+^ EVs that contain CD147 have been detected in colorectal cancer patient sera (Yoshioka et al., 2014). Because microvesicles directly derive from the plasma membrane, it might be expected that the membrane protein repertoire of these EVs reflects that of the plasma membrane. Intriguingly, our study found that the distribution of CD147 and CD98 on EVs is almost mutually exclusive, whereas these proteins have been shown to interact on the cell surface (Cho et al., 2001). Taken together, these findings imply that clustering of CD147 and CD98 on the plasma membrane is dynamically reorganized during microvesicle formation. Along the same lines, Del Conde and colleagues found that activated monocytes release microvesicles that contain tissue factor but are deficient in CD45, whereas both proteins are expressed on the cell surface (Del Conde et al., 2005).

EVs are often more highly secreted by cancer cells than by normal cells (Xu et al., 2018), but the origin of EVs in body fluids of cancer patients has not been established. Elevated levels of circulating CD147 have been detected in cancer patients (Łacina et al., 2022; Lee et al., 2016), and both the proteolytically cleaved extracellular domain of CD147 and its full‐length form are shed by cancer cells (Egawa et al., 2006). Our study supports a prior report that full‐length CD147 is shed, at least in part, in microvesicles (Sidhu et al., 2004). Our analysis of EVs secreted by normal cells indicate that the higher secretion of CD147^+^ EVs and CD98^+^ EVs by cancer cells is reflective of the higher cellular expression of CD147 and CD98 in cancer cells. Notably, our study shows that the prevalence of CD147^+^ EVs significantly increases from an early disease stage in OVCA and RCC patients and in xenograft models, and that CD147^+^ EVs predominantly derive from cancer cells. Other studies have detected elevated levels of CD147^+^ EVs in colorectal cancer patients, but the cell‐of‐origin of these EVs was not identified (Tian et al., 2018; Yoshioka et al., 2014). Furthermore, our study identified that the prevalence of tetraspanin^+^ EVs did not significantly differ between cancer patients and healthy individuals, and that the majority of CD9^+^ EVs, the most abundant subpopulation of tetraspanin^+^ EVs in body fluids, derive from non‐cancerous cells. Whereas the majority of previous functional studies of EVs in cancer have defined EVs by tetraspanin expression, our findings raise the possibility that CD147^+^ EVs could significantly account for the biological responses induced by cancer‐derived EVs. Moreover, our findings indicate that assaying CD147^+^ EVs instead of tetraspanin^+^ EVs or total EVs could be useful for detecting early‐stage or small tumours.

Although a vast number of miRNAs have been detected in EVs, several studies have shown that the majority of EVs contain biologically insignificant amounts of miRNA (Albanese et al., 2021; Chevillet et al., 2014; Zhang et al., 2021). One explanation that could reconcile these findings is the existence of a subpopulation of EVs that is selectively enriched in miRNA, but this subpopulation has hitherto not been defined. A significant discovery in our study is that CD147^+^ EVs have a substantially (8–26‐fold) higher miRNA content than tetraspanin^+^ EVs. CD98^+^ EVs were found to have lower miRNA content than CD147^+^ EVs, indicating that enrichment of miRNA is not common to other microvesicles. Our findings indicate that the higher miRNA content in CD147^+^ EVs stems from the selective enrichment of hnRNP A2/B1 in CD147^+^ EVs through its interaction with CD147. It is unclear whether CD147 directly binds to hnRNP A2/B1. CD147 might interact with hnRNP A2/B1 through caveolin‐1. This possibility is supported by reports that hnRNP A2/B1 mediates sorting of miRNA into microvesicles by interacting with caveolin‐1 (Lee et al., 2019), and that caveolin‐1 interacts with CD147 (Tang & Hemler 2004). Although hnRNP A2/B1 has been reported to control sorting of miRNAs into exosomes (Villarroya‐Beltri et al., 2013), our findings support those of Jeppesen and colleagues who similarly did not detect hnRNP A2/B1 in tetraspanin^+^ EVs (Jeppesen et al., 2019). These investigators recently identified an intriguing class of miRNA‐enriched extracellular nanoparticles termed supermeres that contain hnRNP A2/B1 (Zhang et al., 2021). In contrast to EVs, supermeres lack an encompassing membrane and are substantially smaller than CD147^+^ EVs (i.e. <30 nm in diameter) (Zhang et al., 2021). Furthermore, supermeres are enriched in the RNA‐binding protein AGO2 (Zhang et al., 2021) which we did not detect in CD147^+^ EVs (Figure 5d). In addition, whereas supermeres contain shed ectodomains of membrane proteins (Zhang et al., 2021), only the full‐length form of CD147 was detected in EVs in the present study (Figures 1c and 5d). Collectively, these findings support the notion that CD147^+^ EVs are distinct from supermeres.

Our findings that CD147 is a surface marker of cancer cell‐derived EVs, and that miRNAs are enriched in CD147^+^ EVs, are highly significant for liquid biopsy. Circulating miRNAs have attracted substantial interest as candidate biomarkers for cancer diagnosis, prognosis and recurrence (Dias et al., 2017; Hannafon et al., 2016; Shin et al., 2021; Wang et al., 2022; Zhou et al., 2014). However, trace amounts of miRNAs derived from small tumours may evade detection when isolated from body fluids by conventional methods of extracting all cell‐free miRNA or using polymer‐based reagents that precipitate EVs and also non‐EV material (Théry et al., 2018). Recently, small amounts of EV‐associated miRNAs in sera of breast cancer patients have been detected in situ by using nanoparticle probes called nanoflares (Zhao et al., 2020). However, this approach alone cannot distinguish EV‐associated miRNAs that derive from cancer cells from those that derive from non‐cancerous cells. Our study demonstrates that isolating circulating miRNAs by CD147 immunocapture increases the sensitivity of detecting cancer cell‐specific miRNAs, and that circulating miRNAs isolated by CD147 immunocapture more closely reflect the tumour miRNA signature than circulating miRNAs isolated by conventional methods. However, validation in large cohorts is needed. Because CD147 is overexpressed in ∼20 different types of cancer (Xin et al., 2016; Xiong et al., 2014), CD147 immunocapture could potentially be used to detect cancer‐derived circulating miRNAs in multiple disease sites. Furthermore, this approach only requires a small fluid sample (∼200 μL), does not require specialized equipment, and could be readily utilized in a clinical laboratory.

## AUTHOR CONTRIBUTIONS

Song Yi Ko and Honami Naora developed the original hypothesis, conceived the study, and designed experiments. Song Yi Ko and WonJae Lee performed experiments. Song Yi Ko and Honami Naora analysed data. Melanie Weigert, Eric Jonasch and Ernst Lengyel provided clinical biospecimens. Song Yi Ko and Honami Naora wrote and edited the manuscript. WonJae Lee, Melanie Weigert, Eric Jonasch and Ernst Lengyel edited the manuscript. Honami Naora supervised the study.

## CONFLICT OF INTEREST STATEMENT

The authors declare no competing interests.

## Supporting information

Supporting Information

Supporting Information

## Data Availability

All data associated with this study are present in the article and the Supplementary Information.
