# Supplementary material for: The glycoprotein CD147 defines miRNA‐enriched extracellular vesicles that derive from cancer cells
Source: J Extracell Vesicles. 2023 Mar 27;12(4):12318. doi: 10.1002/jev2.12318 (PMC10042814; doi:10.1002/jev2.12318)
Supplement: Supplementary file 1 — Supporting Information [file JEV2-12-12318-s001.pdf]

Title:

The glycoprotein CD147 defines miRNA-enriched extracellular vesicles that derive from cancer cells

Authors:

Song Yi Ko, WonJae Lee, Melanie Weigert, Eric Jonasch, Ernst Lengyel, Honami Naora

SUPPLEMENTARY FIGURES

Fig. S1:	Cellular expression of candidate surface markers
Fig. S2:	Size distribution of purified EVs
Fig. S3:	Detection of surface markers in EVs by flow cytometry
Fig. S4.	Experimental scheme and analysis of EV depletion
Fig. S5:	Expression of CD147 and CD98 in tetraspanin-negative EVs
Fig. S6:	miRNA content in EV subpopulations
Fig. S7:	Expression of EV surface markers and distribution of EV subpopulations in hnRNP A2/B1-knockout cells
Fig. S8.	Cellular expression of CD147 and CD98 and distribution of EV subpopulations in normal cells
Fig. S9:	Detection of surface markers by species-specific antibodies
Fig. S10:	Prevalence of EV subpopulations in plasma of patients with benign gynecologic conditions and with OVCA
Fig. S11:	Prevalence of EV subpopulations in plasma of patients with RCC
Fig. S12:	Comparison of methods to isolate cancer-associated miRNAs from body fluids

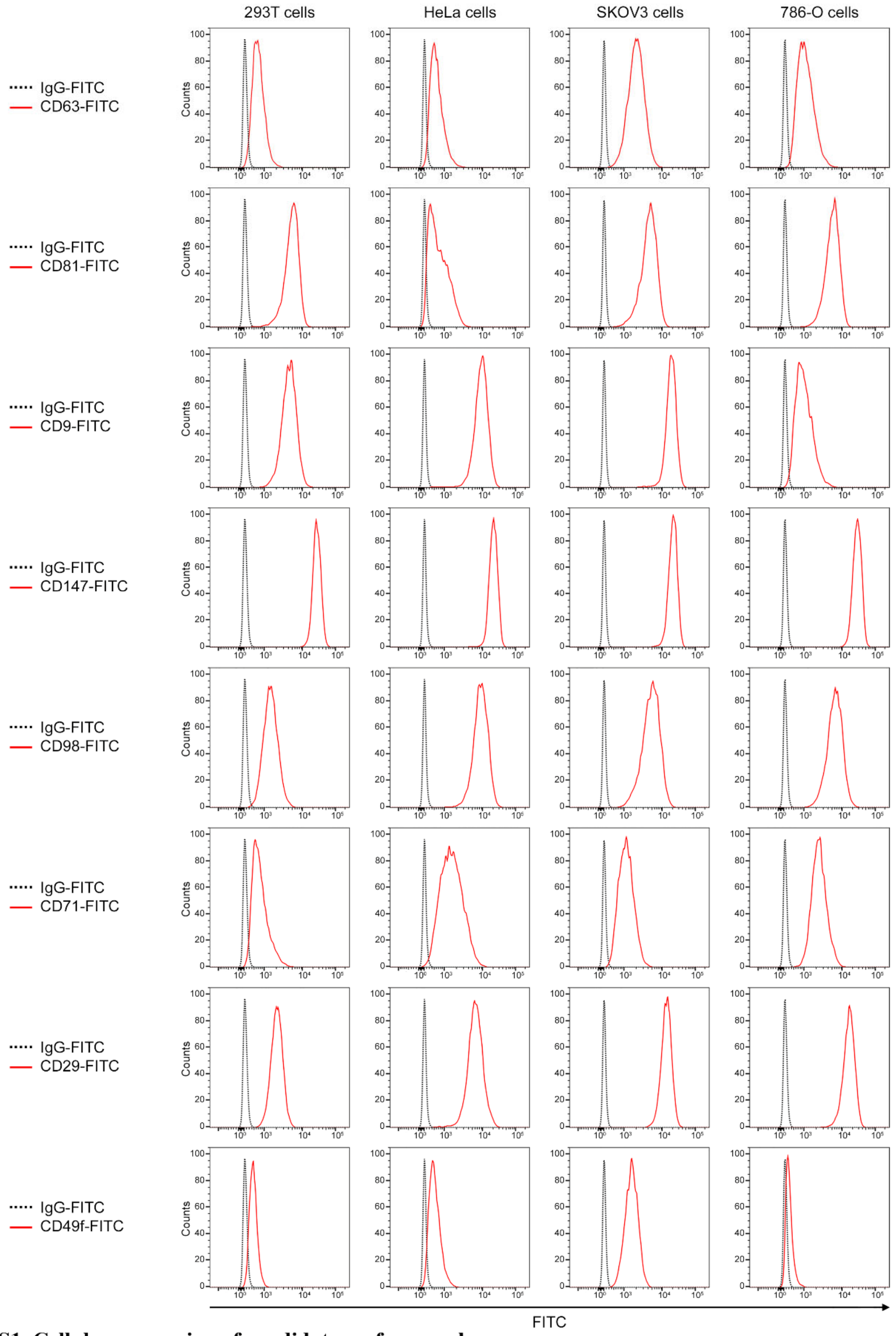

Figure S1. Cellular expression of candidate surface markers.
Expression of common membrane proteins in the top 100 proteins in the ExoCarta and Vesiclepedia databases was evaluated in 293T, HeLa, SKOV3 and 786-O cells by flow cytometry. A minimum of 10,000 events in the gated population of viable singlet cells were analyzed for each sample. Three independent experiments were performed to verify expression of each protein in each cell type. Shown are representative histogram plots of staining with FITC-conjugated antibody to the indicated protein (red line) and with IgG isotype control (dotted line). ATP1A1 and CLIC1 were excluded from analysis because the lack of commercially available fluorochrome-conjugated antibodies to these proteins precluded further study of these proteins in EVs.

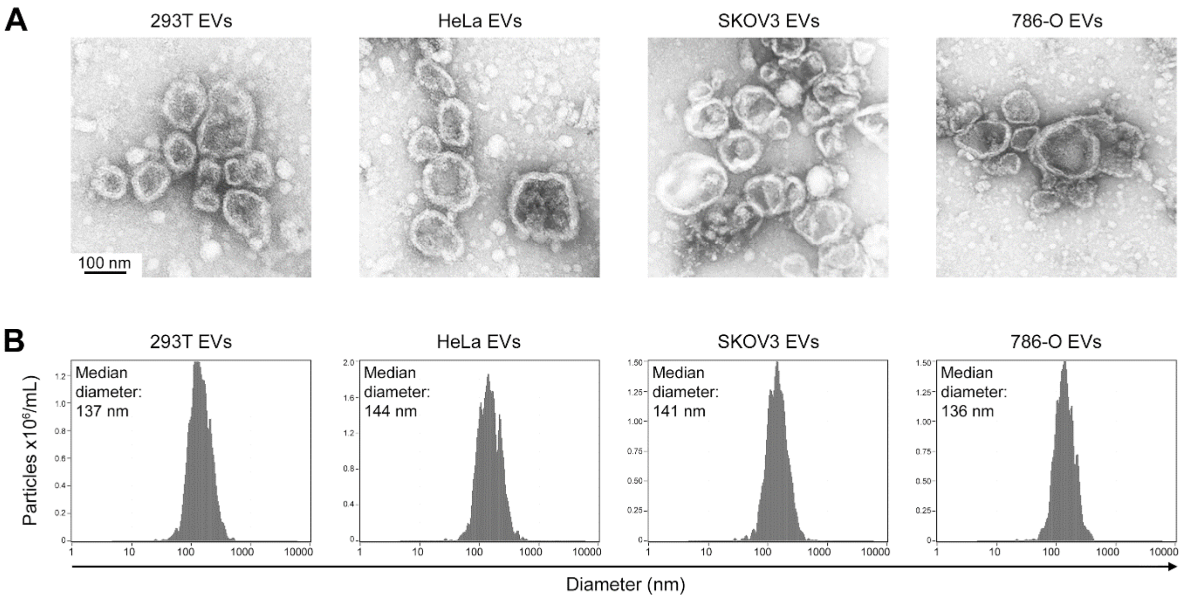

Figure S2. Size distribution of purified EVs.
(**A**) Visualization of purified EVs by transmission electron microscopy. (**B**) Evaluation of size distribution of purified EVs by nanoparticle tracking analysis. Each plot shows the combined result of 10 replicate measurements. Median diameters of EVs are indicated.

A

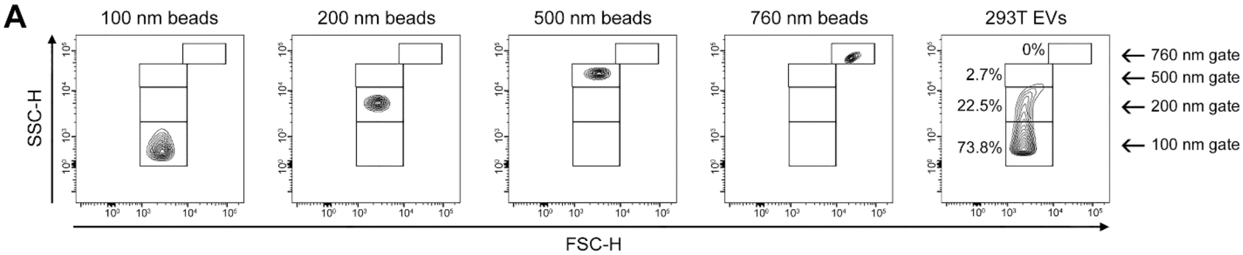

Figure S3. Detection of surface markers in EVs by flow cytometry.
(**A**) Flow cytometry settings were optimized for EV detection by acquiring microbeads of different diameters (100 nm, 200 nm, 500 nm, 760 nm). Shown are representative forward scatter versus side scatter plots of the bead populations, and of 293T cell-derived EVs that were acquired using the same settings. Estimates of size distribution of EVs based on the size marker bead gates are indicated. (**B**) EVs derived from 293T, HeLa, SKOV3 and 786-O cells were directly stained with FITC-conjugated antibodies to CD63, CD81, CD9, CD147, CD98, CD71, CD29 and CD49f and with IgG isotype control, and evaluated by flow cytometry. A minimum of 10,000 gated singlet EVs were analyzed for each sample. Three independent experiments were performed to verify expression of each marker in EVs derived from each cell type, where each experiment used a different batch of EVs. Shown are representative plots of staining, indicating the percentage of EVs that express a given surface marker.

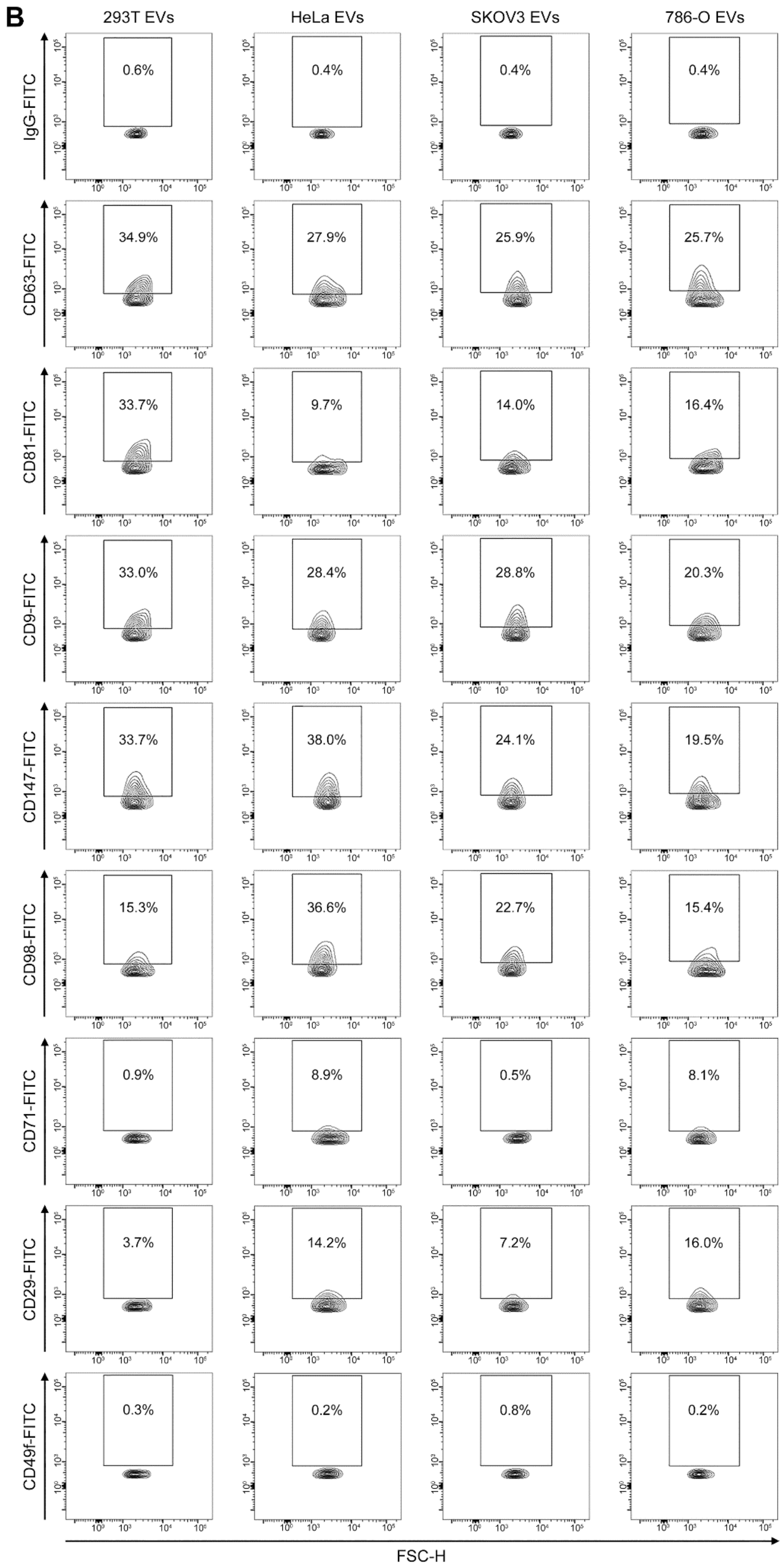
B
293T EVs
HeLa EVs
SKOV3 EVs
786-O EVs
IgG-FITC
0.6%
0.4%
0.4%
0.4%
CD63-FITC
34.9%
27.9%
25.9%
25.7%
CD81-FITC
33.7%
9.7%
14.0%
16.4%
CD9-FITC
33.0%
28.4%
28.8%
20.3%
CD147-FITC
33.7%
38.0%
24.1%
19.5%
CD98-FITC
15.3%
36.6%
22.7%
15.4%
CD71-FITC
0.9%
8.9%
0.5%
8.1%
CD29-FITC
3.7%
14.2%
7.2%
16.0%
CD49f-FITC
0.3%
0.2%
0.8%
0.2%
FSC-H

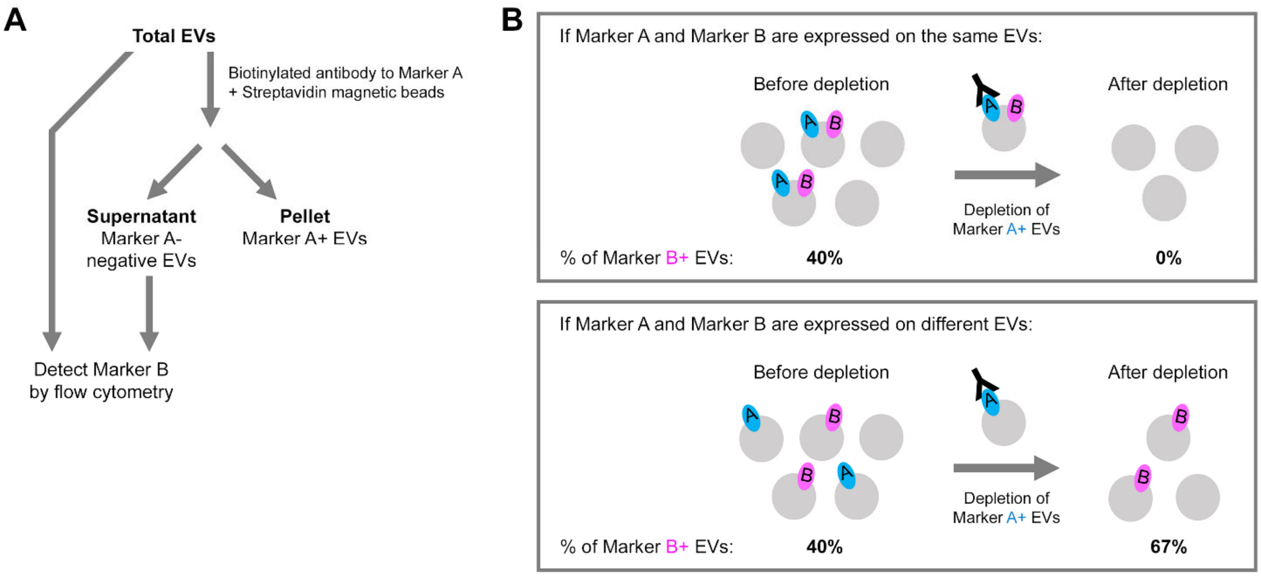

Figure S4. Experimental scheme and analysis of EV depletion.
(**A**) Experimental scheme in which batches of total EVs were depleted of EVs that express a given surface marker (Marker A) by immunocapture. The percentage of EVs that express another given surface marker (Marker B) was evaluated by flow cytometry in the remaining pool of Marker A-negative EVs (post-depletion) and in the original total EV population (pre-depletion). (**B**) Examples of expected percentages of EVs that express Marker B prior to and following depletion of EVs that express Marker A, where Marker A and Marker B are expressed on the same EVs and where Marker A and Marker B are expressed on different EVs.

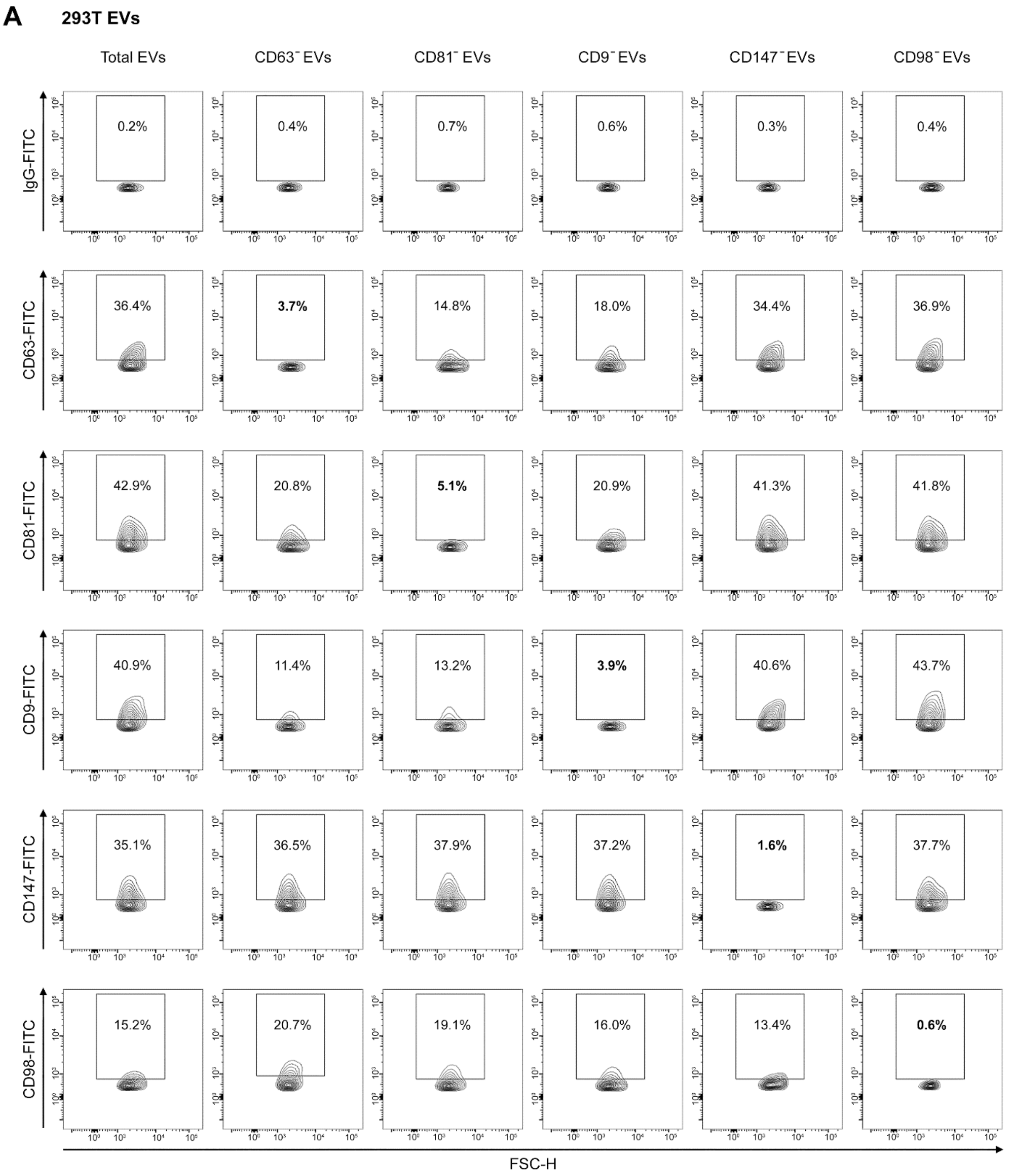

Figure S5. Expression of CD147 and CD98 in tetraspanin-negative EVs.

(**A-E**) Batches of total EVs were depleted of EVs that express a given surface marker (Marker A). The remaining pool of EVs (Marker A-negative subpopulation) and the total EV population were assayed for other markers (refer Figure S4A). Shown are representative plots of staining of total EVs and subpopulations of EVs derived from 293T (**A**), HeLa (**B**), SKOV3 (**C**) and 786-O (**D**) cells with FITC-conjugated antibodies to CD63, CD81, CD9, CD147 and CD98 and with IgG isotype control. A minimum of 10,000 gated singlet EVs were analyzed for each sample. Percentages of EVs that express a given marker in the total EV population and in each subpopulation of marker-negative EVs are indicated. (**E**) Mean ± SD of $n = 3$ independent experiments using EVs derived from HeLa, SKOV3 and 786-O cells, where each experiment used a different batch of EVs. Data of EVs derived from 293T cells is shown in Figure 1D. (**F**) Total EVs derived from 293T cells were depleted of $CD63^+$ EVs, $CD81^+$ EVs and $CD9^+$ EVs (triple depletion) or depleted using Ig isotype controls (negative control). Shown are percentages of EVs that express the indicated markers following depletion (mean ± SD of $n = 4$ independent experiments). ns, not significant, *$P < 0.05$, **$P < 0.01$, ***$P < 0.001$, ****$P < 0.0001$ by one-way ANOVA with Bonferroni's corrections in **E**; by unpaired two-tailed Student's *t*-test in **F**.

B

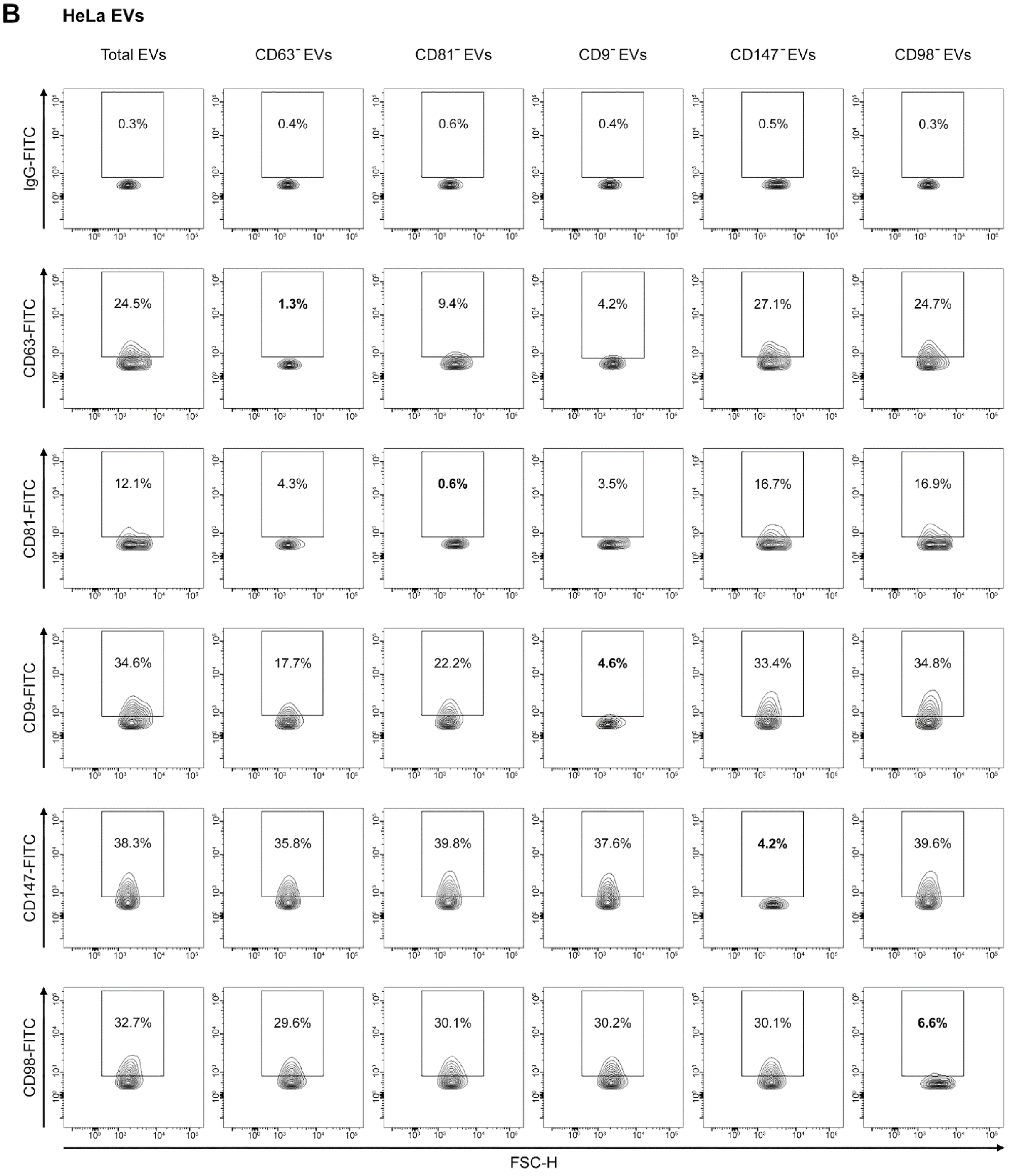

HeLa EVs
Total EVs
CD63⁻ EVs
CD81⁻ EVs
CD9⁻ EVs
CD147⁻ EVs
CD98⁻ EVs
IgG-FITC
0.3%
0.4%
0.6%
0.4%
0.5%
0.3%
CD63-FITC
24.5%
1.3%
9.4%
4.2%
27.1%
24.7%
CD81-FITC
12.1%
4.3%
0.6%
3.5%
16.7%
16.9%
CD9-FITC
34.6%
17.7%
22.2%
4.6%
33.4%
34.8%
CD147-FITC
38.3%
35.8%
39.8%
37.6%
4.2%
39.6%
CD98-FITC
32.7%
29.6%
30.1%
30.2%
30.1%
6.6%
FSC-H

C **SKOV3 EVs**

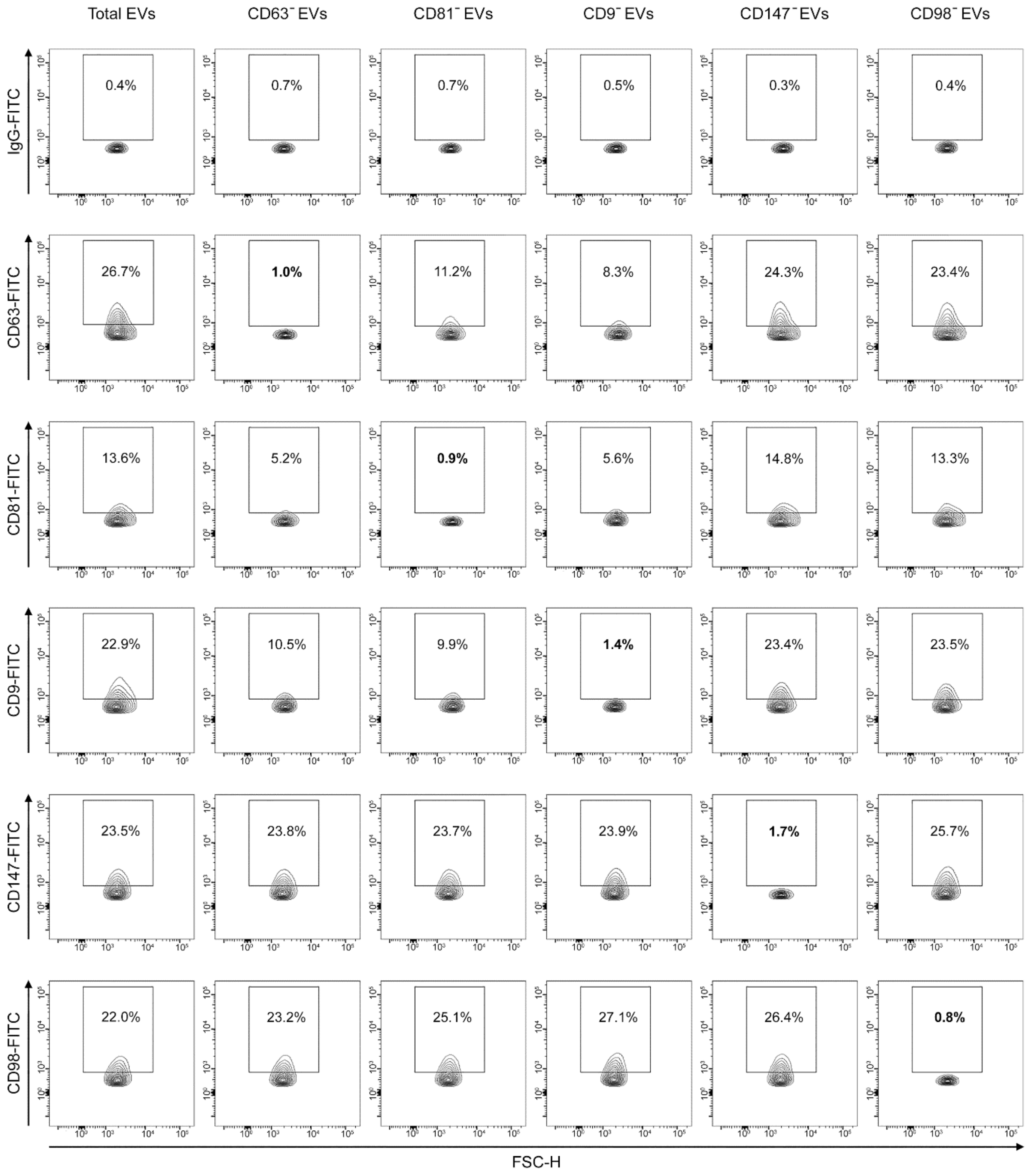
Total EVs
CD63^{-} EVs
CD81^{-} EVs
CD9^{-} EVs
CD147^{-} EVs
CD98^{-} EVs
IgG-FITC
0.4%
0.7%
0.7%
0.5%
0.3%
0.4%
CD63-FITC
26.7%
1.0%
11.2%
8.3%
24.3%
23.4%
CD81-FITC
13.6%
5.2%
0.9%
5.6%
14.8%
13.3%
CD9-FITC
22.9%
10.5%
9.9%
1.4%
23.4%
23.5%
CD147-FITC
23.5%
23.8%
23.7%
23.9%
1.7%
25.7%
CD98-FITC
22.0%
23.2%
25.1%
27.1%
26.4%
0.8%
FSC-H

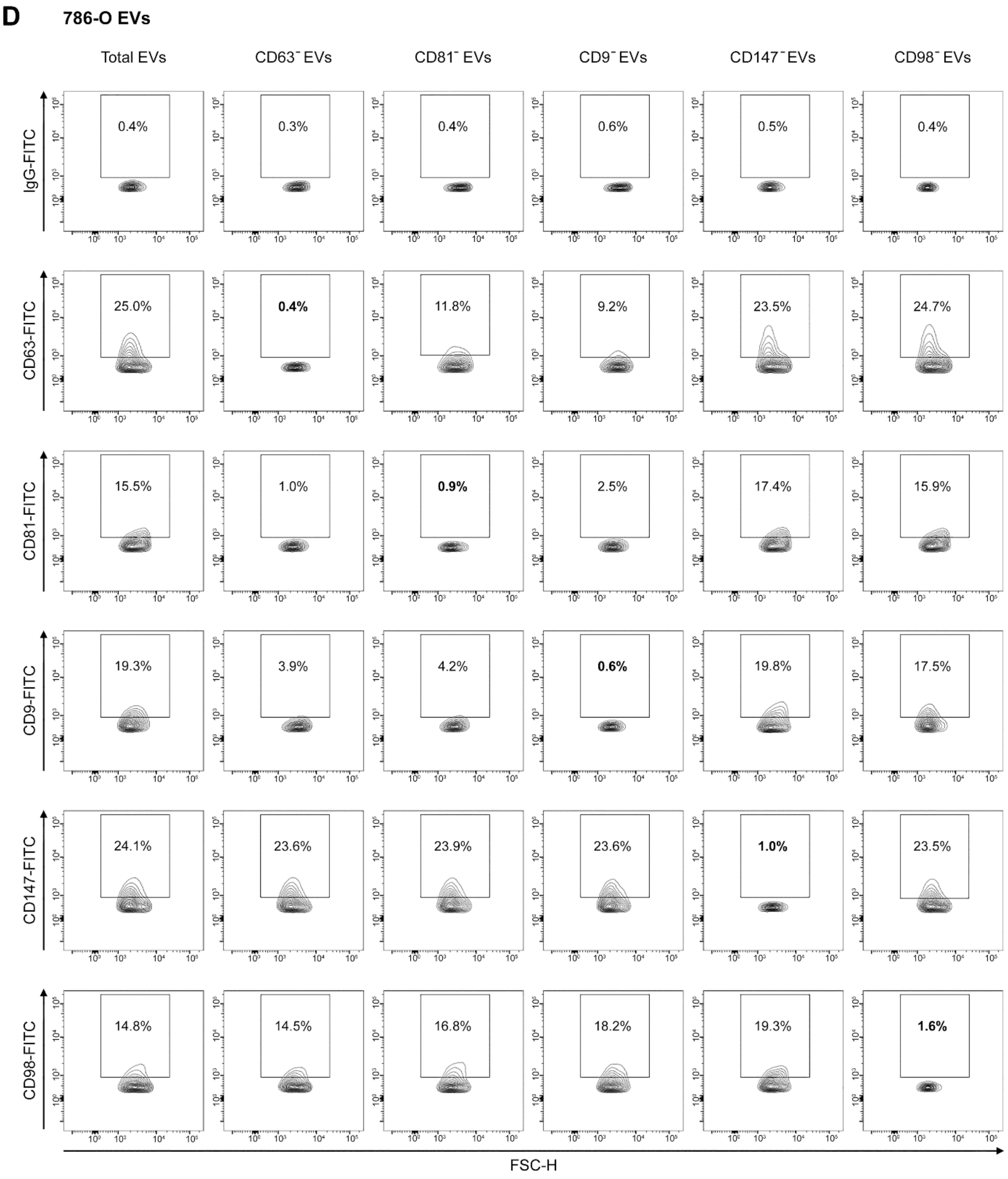
D
786-O EVs
Total EVs
CD63⁻ EVs
CD81⁻ EVs
CD9⁻ EVs
CD147⁻ EVs
CD98⁻ EVs
IgG-FITC
0.4%
0.3%
0.4%
0.6%
0.5%
0.4%
CD63-FITC
25.0%
0.4%
11.8%
9.2%
23.5%
24.7%
CD81-FITC
15.5%
1.0%
0.9%
2.5%
17.4%
15.9%
CD9-FITC
19.3%
3.9%
4.2%
0.6%
19.8%
17.5%
CD147-FITC
24.1%
23.6%
23.9%
23.6%
1.0%
23.5%
CD98-FITC
14.8%
14.5%
16.8%
18.2%
19.3%
1.6%
FSC-H

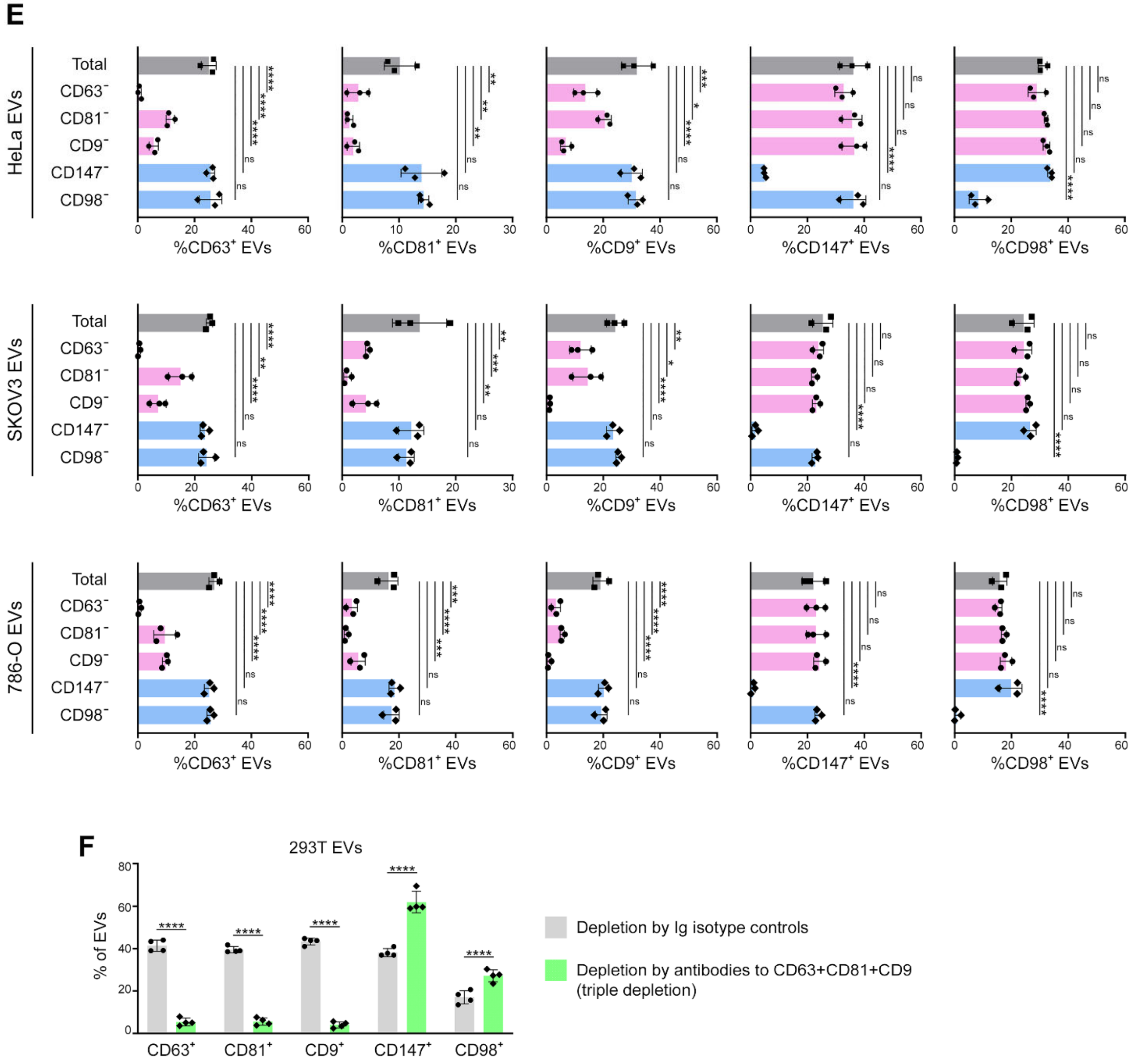
E
HeLa EVs
SKOV3 EVs
786-O EVs
Total
CD63⁻
CD81⁻
CD9⁻
CD147⁻
CD98⁻
%CD63⁺ EVs
%CD81⁺ EVs
%CD9⁺ EVs
%CD147⁺ EVs
%CD98⁺ EVs
F
293T EVs
% of EVs
CD63⁺
CD81⁺
CD9⁺
CD147⁺
CD98⁺
Depletion by Ig isotype controls
Depletion by antibodies to CD63+CD81+CD9 (triple depletion)

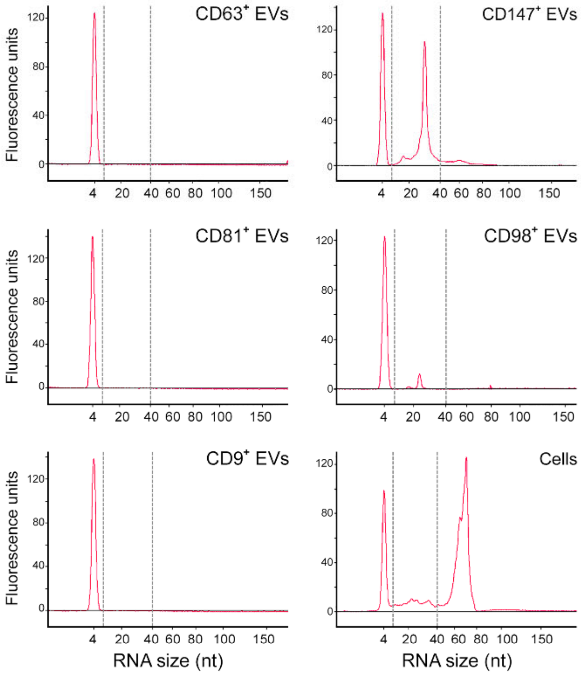

Figure S6. miRNA content in EV subpopulations.
Agilent 2100 Bioanalyzer™ electropherogram profiles of small RNA isolated from 293T cells and from comparable numbers of 293T cell-derived EVs of each indicated subpopulation (~2 x 10^7). Small RNAs detected between the two dotted lines were considered as miRNAs. The peak at 4 nt corresponds to the loading control.

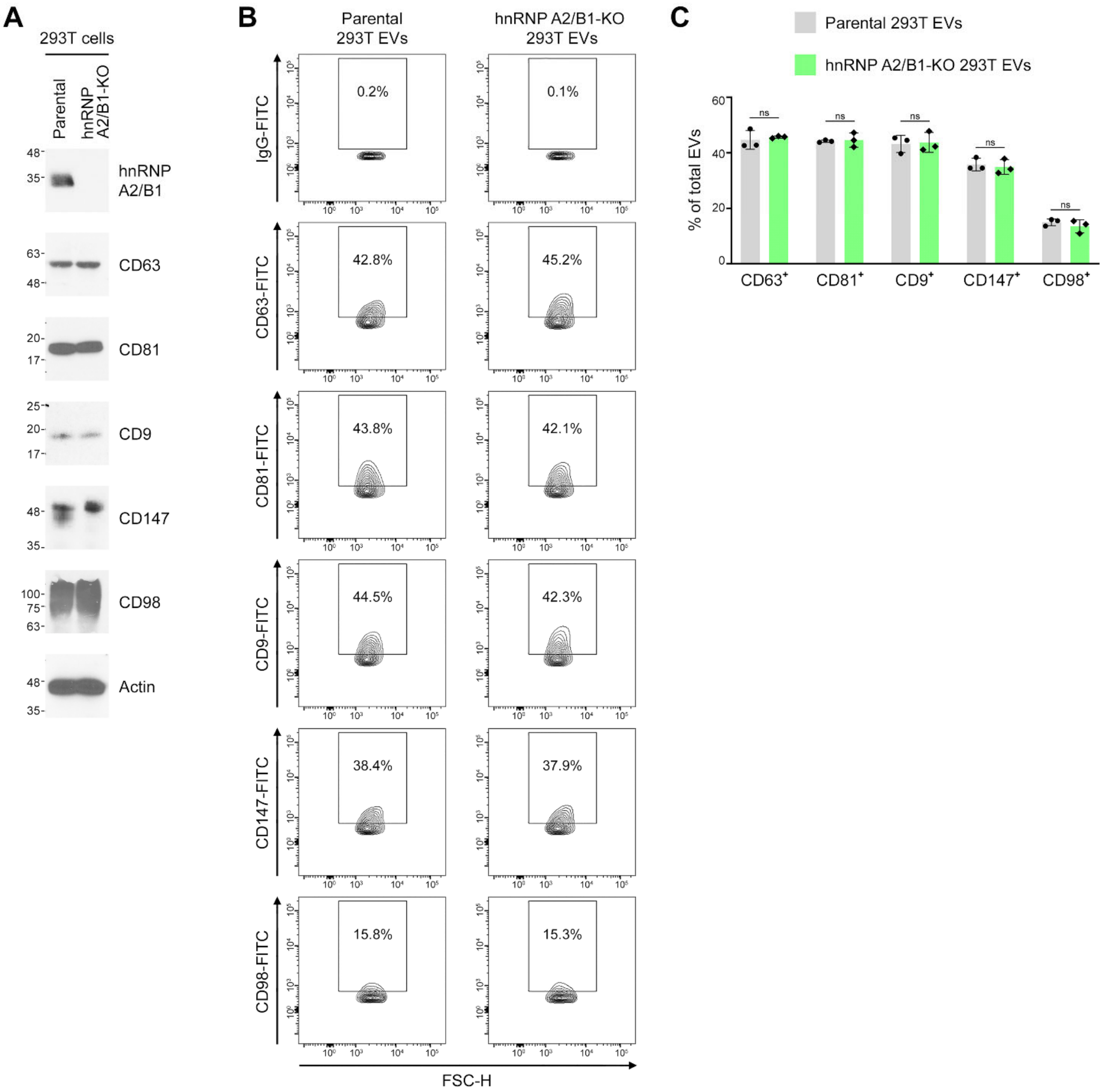

Figure S7. Expression of EV surface markers and distribution of EV subpopulations in hnRNP A2/B1-knockout cells.

(**A**) Immunoblot of hnRNP A2/B1 in equivalent amounts of cell lysates (20 µg) of parental 293T cells and 293T cells in which the *HNRNPA2B1* gene was deleted by CRISPR/Cas9 gene editing (hnRNP A2/B1-KO). Cell lysates were also evaluated for the indicated surface markers to confirm that cellular expression levels of these markers are not affected by knockout of hnRNP A2/B1. (**B**, **C**) Expression of surface markers in EVs derived from parental and hnRNP A2/B1-KO 293T cells. In (**B**), representative contour plots of staining of EVs with FITC-conjugated antibodies to CD63, CD81, CD9, CD147 and CD98 and with IgG isotype control. A minimum of 10,000 gated singlet EVs were analyzed for each sample. Percentages of EVs that express a given marker are indicated. In (**C**), mean ± SD of $n = 3$ independent experiments where each experiment used a different batch of EVs. ns, not significant, by unpaired two-tailed Student's *t*-test in **C.**

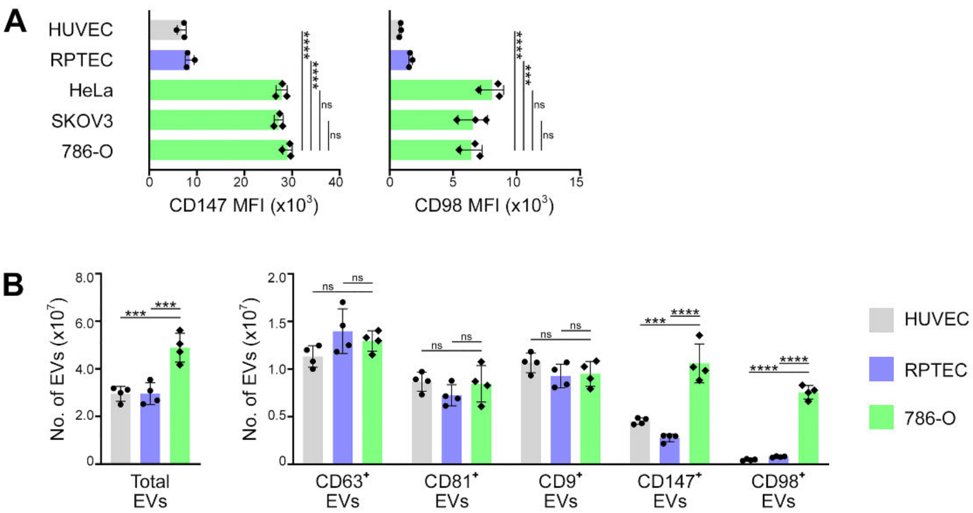

Figure S8. Cellular expression of CD147 and CD98 and distribution of EV subpopulations in normal cells. (**A**) Mean fluorescence intensity (MFI) of staining of CD147 and CD98 detected by flow cytometry in normal primary human umbilical vein endothelial cells (HUVEC), normal primary human renal proximal tubule epithelial cells (RPTEC) and the indicated cancer cell lines. A minimum of 10,000 events in the gated population of viable singlet cells were analyzed for each sample. Mean ± SD of $n = 3$ independent experiments are shown. (**B**) Numbers of total EVs and numbers of CD63$^+$, CD81$^+$, CD9$^+$, CD147$^+$ and CD98$^+$ EVs secreted by equivalent numbers of HUVEC, RPTEC and 786-O RCC cells (~5 x 10^6). Mean ± SD of $n = 4$ independent experiments are shown. ns, not significant, ***$P < 0.001$, ****$P < 0.0001$ by one-way ANOVA with Bonferroni's corrections in **A** and **B.**

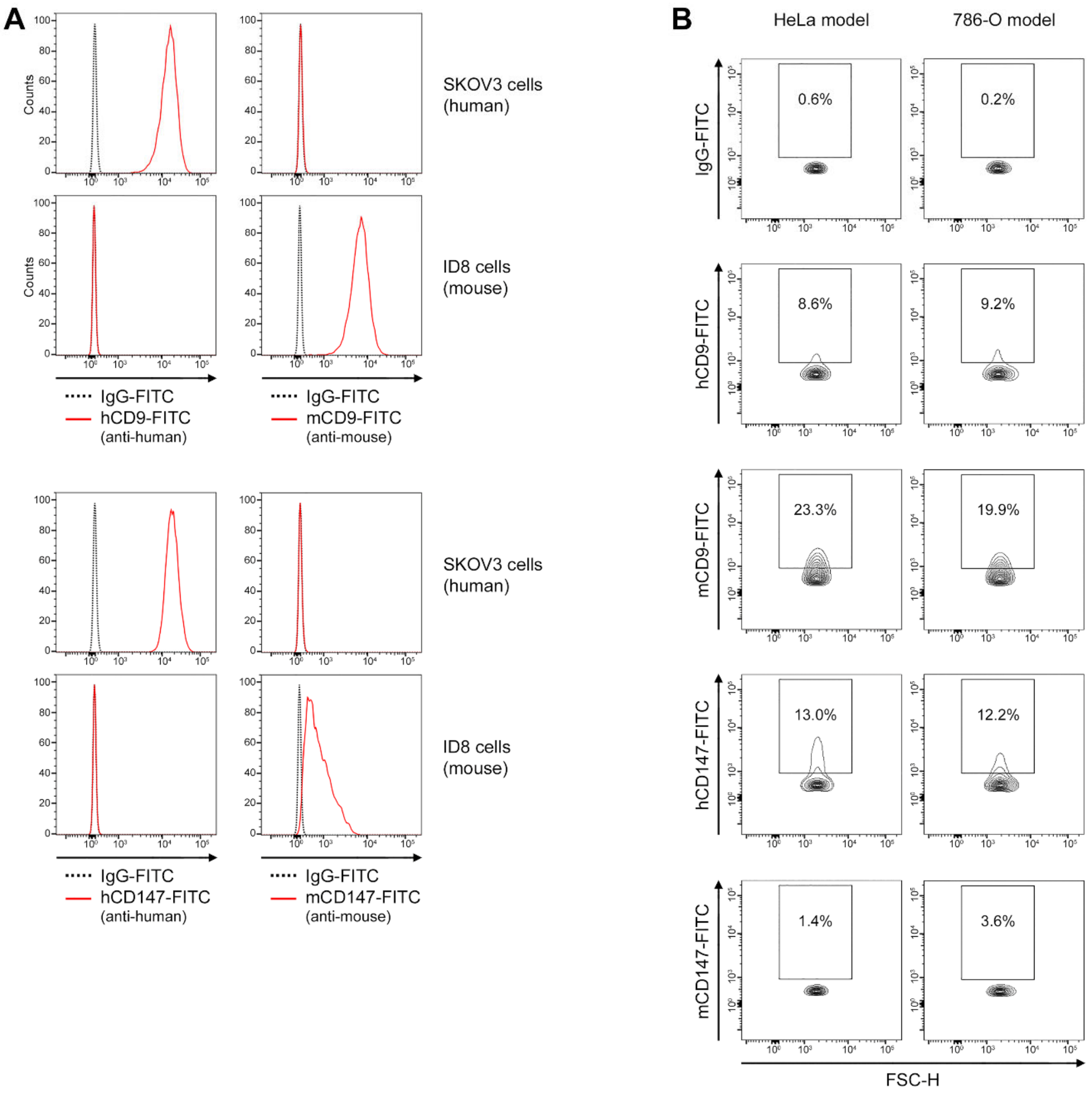

Figure S9. Detection of surface markers by species-specific antibodies.
(**A**) Specificity of antibodies to human and mouse CD9, and to human and mouse CD147, was confirmed by staining SKOV3 human ovarian cancer cells and ID8 mouse ovarian cancer cells. A minimum of 10,000 events in the gated population of viable singlet cells were analyzed for each sample. Shown are representative histogram plots of staining with the indicated FITC-conjugated antibodies to CD9 and CD147 (red line) and with IgG isotype control (dotted line). (**B**) Plasma was collected from mice with s.c. tumors derived from HeLa cells (at Day 50) or from 786-O cells (at Day 49) (refer Figure 6A). Plasma EVs that derive from human cancer cells and from non-cancerous mouse host cells were distinguished by staining with antibodies specific to human and mouse markers. A minimum of 10,000 gated singlet EVs were analyzed for each sample. Shown are representative contour plots of staining, indicating the percentage of EVs that express a given surface marker.

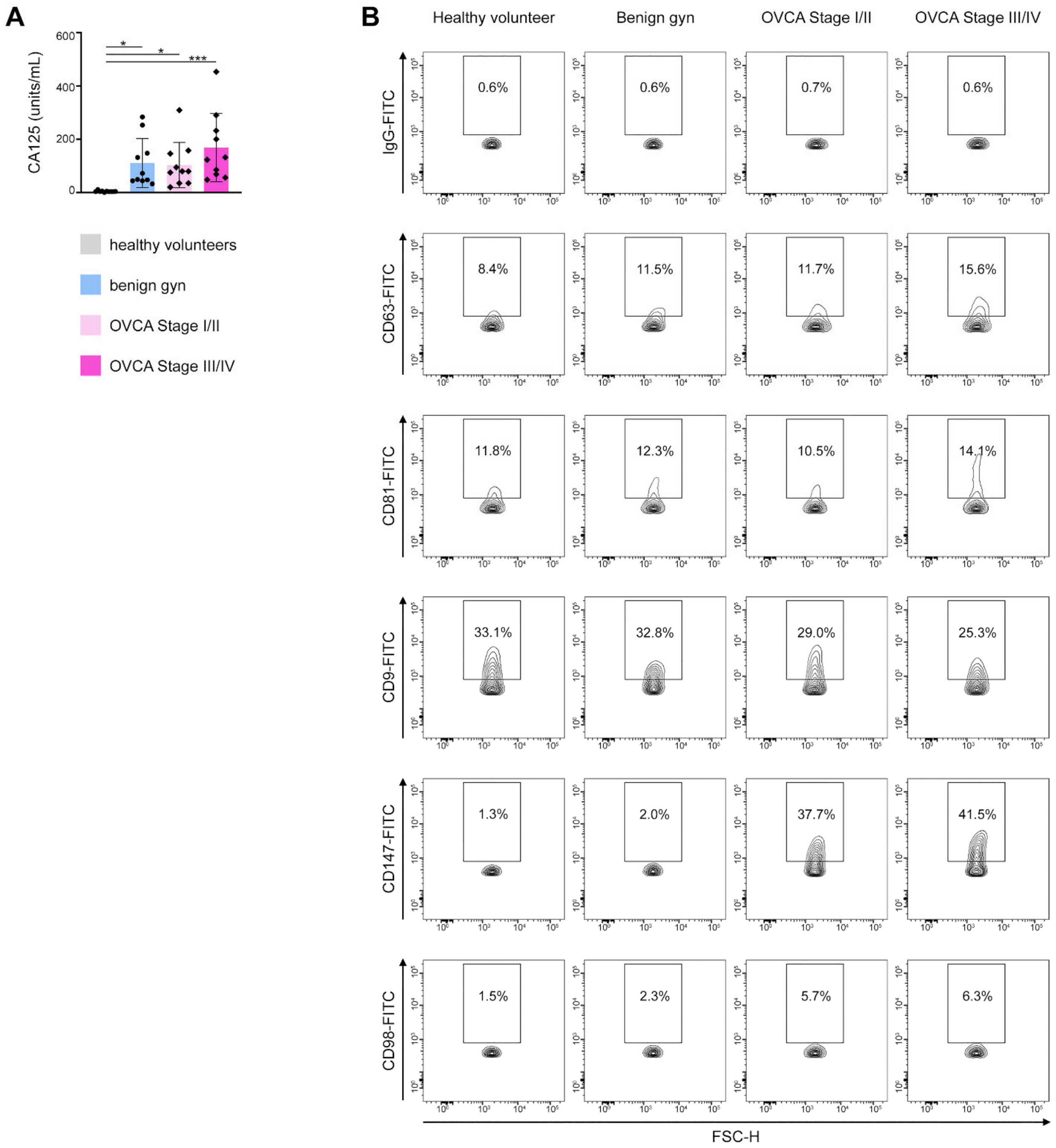

Figure S10. Prevalence of EV subpopulations in plasma of patients with benign gynecologic conditions and with OVCA.

(**A**) CA125 levels in plasma of healthy adult volunteers and patients with either benign gynecologic (gyn) conditions, Stage I/II OVCA or Stage III/IV OVCA, evaluated by ELISA (n =10 cases per group). Clinicopathologic features of cases are described in Table S2. (**B**) EVs were isolated from plasma of each case, stained with FITC-conjugated antibodies to CD63, CD81, CD9, CD147 and CD98 and with IgG isotype control, and evaluated by flow cytometry. A minimum of 10,000 gated singlet EVs were analyzed for each sample. Shown are representative contour plots of staining, indicating the percentage of EVs that express a given surface marker. $*P < 0.05$, $***P < 0.001$ by one-way ANOVA with Bonferroni's corrections in **A**.

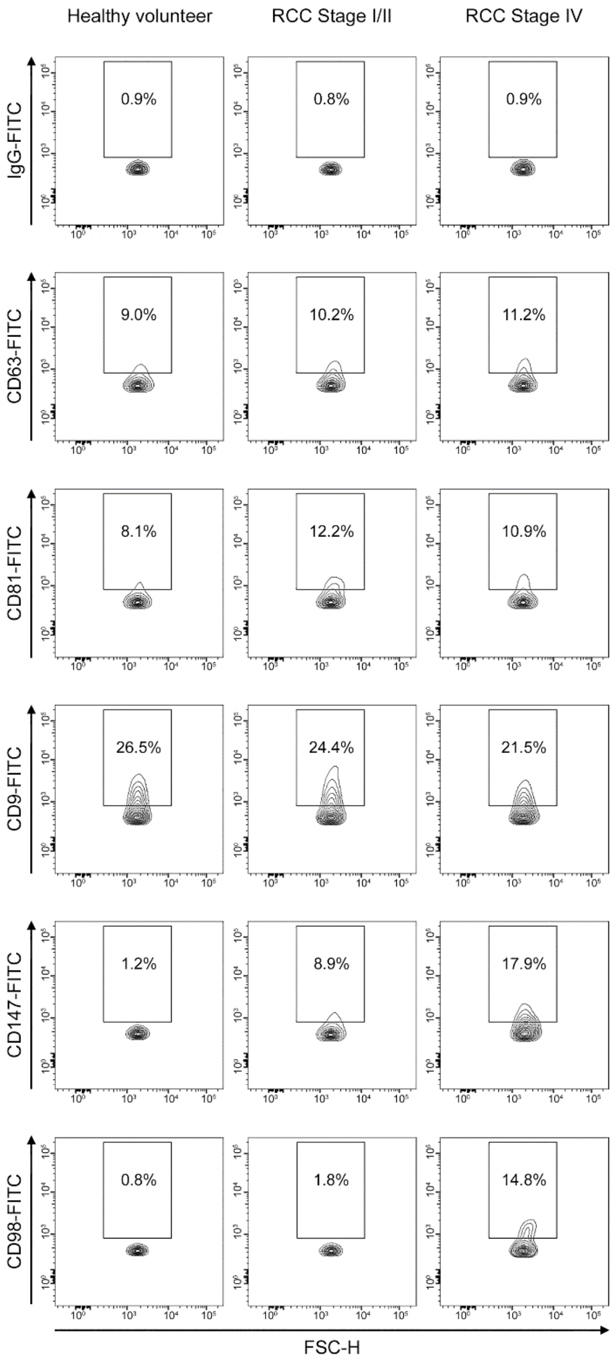

Figure S11. Prevalence of EV subpopulations in plasma of patients with RCC.
EVs were isolated from plasma of healthy adult volunteers and patients with either Stage I/II RCC or Stage IV RCC. Clinicopathologic features of cases are described in Table S2. Plasma EVs of each case were stained with FITC-conjugated antibodies to CD63, CD81, CD9, CD147 and CD98 and with IgG isotype control, and evaluated by flow cytometry. A minimum of 10,000 gated singlet EVs were analyzed for each sample. Shown are representative contour plots of staining, indicating the percentage of EVs that express a given surface marker.

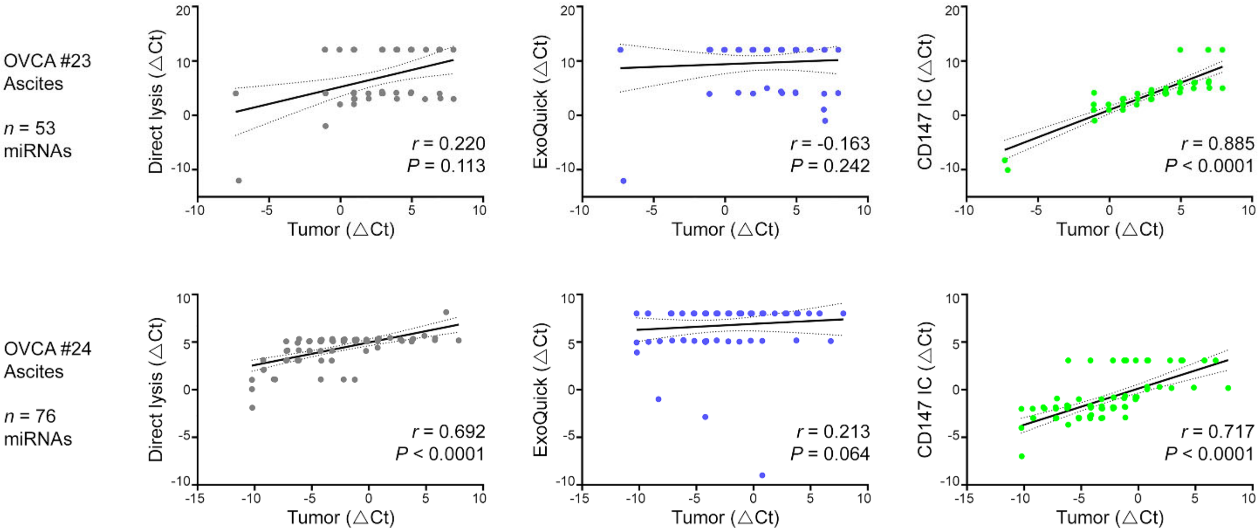

Figure S12. Comparison of methods to isolate cancer-associated miRNAs from body fluids.
miRNA was isolated from equivalent volumes of ascites (200 µL) of OVCA patients by either direct lysis of whole ascites, precipitation using ExoQuick® reagent, or immunocapture (IC) with CD147 antibody. Expression levels of 84 cancer-associated miRNAs in each of the three ascites-derived samples of each case were evaluated by using a miRCURY LNA™ miRNA cancer focus PCR panel (Qiagen). For each case, only those miRNAs that were detected in at least one ascites-derived sample were considered for correlation analysis. Shown are Spearman rank correlations between levels of miRNAs in each ascites-derived sample and levels of miRNAs in matching tumor tissue of each case. Dotted lines indicate 95% confidence intervals. The number of miRNAs analyzed for each case is indicated.
