# Supplementary material for: The glycoprotein CD147 defines miRNA‐enriched extracellular vesicles that derive from cancer cells
Source: J Extracell Vesicles. 2023 Mar 27;12(4):12318. doi: 10.1002/jev2.12318 (PMC10042814; doi:10.1002/jev2.12318)
Supplement: Supplementary file 2 — Supporting Information [file JEV2-12-12318-s002.pdf]

Title:

The glycoprotein CD147 defines miRNA-enriched extracellular vesicles that derive from cancer cells

Authors:

Song Yi Ko, WonJae Lee, Melanie Weigert, Eric Jonasch, Ernst Lengyel, Honami Naora

SUPPLEMENTARY TABLES

Table S1: Sources and concentrations of antibodies

Table S2: Clinicopathologic features of cases

Table S1: Sources and concentrations of antibodies

Reagent	Source	Identifier	Working concentration
Antibodies for flow cytometry and immunocytochemistry			
FITC-Human CD63	BioLegend	353008	1μg/100μL
FITC-Human CD81	BioLegend	349504	1μg/100μL
FITC-Human CD9	BioLegend	312104	1μg/100μL
FITC-Human CD147	BioLegend	306204	1μg/100μL
FITC-Human CD98	BioLegend	315603	1μg/100μL
FITC-Human CD71	BioLegend	334103	1μg/100μL
Alexa Fluor 488-Human CD29	BioLegend	303015	1μg/100μL
FITC-Human CD49f	BioLegend	313605	1μg/100μL
FITC-Mouse CD9	BioLegend	124807	1μg/100μL
FITC-Mouse CD147	BioLegend	123705	1μg/100μL
FITC Mouse IgG1 isotype control	BioLegend	400107	1μg/100μL
FITC Mouse IgG2a isotype control	BioLegend	400207	1μg/100μL
FITC Rat IgG2a isotype control	BioLegend	400505	1μg/100μL
Alexa Fluor 488 Mouse IgG1 isotype control	BioLegend	400132	1μg/100μL
Antibodies for immunoblot and immunoprecipitation			
TSG101	Abcam	ab125011	0.2 μg/mL
HGS (HRS)	Cell Signaling Technology	15087	1:1000
CD63	Santa Cruz Biotechnology	sc-15363	0.4 μg/mL
CD81	Santa Cruz Biotechnology	sc-166029	0.4 μg/mL
CD9	Cell Signaling Technology	13403	1:1000
CD147	Cell Signaling Technology	13287	1:1000
CD98	Cell Signaling Technology	47213	1:1000
hnRNP A2/B1	Abcam	ab31645	1 μg/mL
APOA1	Cell Signaling Technology	3350	1:1000
AGO2	Cell Signaling Technology	2897	1:1000
HRP-conjugated secondary antibodies	Cell Signaling Technology	7076; 7074	1:2000
Antibodies for immunogold labeling			
CD63	BioLegend	353039	20 μg/mL
CD81	BioLegend	349502	20 μg/mL
CD9	BioLegend	312102	20 μg/mL
CD147	BioLegend	306202	20 μg/mL
CD98	BioLegend	315602	20 μg/mL
10nm goat-anti-mouse IgG gold conjugate	Electron Microscopy Sciences	25129	1:20
Antibodies for EV immunocapture			
Biotinylated anti-CD63	Abcam	ab134331	10μg/100μL bead slurry
Biotinylated anti-CD81	Abcam	ab239238	10μg/100μL bead slurry
Biotinylated anti-CD9	Abcam	ab28094	10μg/100μL bead slurry
Biotinylated anti-CD147	Abcam	ab21898	10μg/100μL bead slurry
Biotinylated anti-CD98	Abcam	ab26009	10μg/100μL bead slurry

Table S2: Clinicopathologic features of cases

Group	Case	Pathology	Disease Stage
**Benign gynecologic conditions***	BG #1	Endometriosis	
	BG #2	Endometriosis	
	BG #3	Endometriosis	
	BG #4	Endometriosis	
	BG #5	Simple ovarian cysts	
	BG #6	Serous cystadenoma/cystadenofibroma	
	BG #7	Mucinous cystadenoma	
	BG #8	Ovarian fibroma	
	BG #9	Ovarian adenofibroma	
	BG #10	Teratoma	
**Ovarian carcinoma***	OVCA #1	Clear cell	IA
	OVCA #2	Endometrioid	IA
	OVCA #3	Mucinous	IC
	OVCA #4	Clear cell	IC
	OVCA #5	Endometrioid	IC
	OVCA #6	Clear cell	IC
	OVCA #7	High-grade with endometrioid and clear cell features	IC
	OVCA #8	Endometrioid	IIA
	OVCA #9	High-grade serous	IIB
	OVCA #10	High-grade serous	IIB
	OVCA #11	Mucinous	III
	OVCA #12	High-grade serous	III
	OVCA #13	High-grade serous	IIIA
	OVCA #14	Clear cell	IIIC
	OVCA #15	Endometrioid	IIIC
	OVCA #16	High-grade serous	IIIC
	OVCA #17	Low-grade serous	IIIC
	OVCA #18	High-grade serous	IVA
	OVCA #19	Clear cell	IVB
	OVCA #20	High-grade serous	IVB
	OVCA #21‡	High-grade serous	IIIC
	OVCA #22‡	High-grade serous	IIIB
	OVCA #23‡	High-grade serous	IIIA
	OVCA #24‡	High-grade serous	IIIC
**Renal cell carcinoma†**	RCC #1	Clear cell	IA
	RCC #2	Clear cell	IA
	RCC #3	Clear cell	IA
	RCC #4	Clear cell	IA
	RCC #5	Clear cell	IA
	RCC #6	Clear cell	IA
	RCC #7	Clear cell	IA
	RCC #8	Clear cell	IB
	RCC #9	Clear cell	IB
	RCC #10	Clear cell	IIA
	RCC #11	Clear cell	IV
	RCC #12	Clear cell	IV
	RCC #13	Clear cell	IV
	RCC #14	Clear cell	IV
	RCC #15	Clear cell	IV
	RCC #16	Clear cell	IV
	RCC #17	Clear cell	IV
	RCC #18	Clear cell	IV
	RCC #19	Clear cell	IV
	RCC #20	Clear cell	IV
	RCC #21‡	Clear cell	IIIA

*Analysis of plasma samples of BG #1 to #10 and OVCA #1 to #20 shown in Figure 7A, B and Figure S10A.
† Analysis of plasma samples of RCC #1 to #20 shown in Figure 7C, D and Figure 8D, E.
‡ Analysis of fluid (plasma or ascites) and matching tumor tissue shown in Figure 8C and Figure S12.
